# Barriers and facilitators for healthcare access among immigrants in Japan: a mixed methods systematic review and meta-synthesis

**DOI:** 10.1016/j.lanwpc.2024.101276

**Published:** 2025-01-10

**Authors:** Yu Par Khin, Floret Maame Owusu, Nobutoshi Nawa, Pamela J. Surkan, Takeo Fujiwara

**Affiliations:** a Department of Public Health, Institute of Science Tokyo, Tokyo, Japan; b Center for Well-being Research Advancement, Institute of Science Tokyo, Japan; c Department of International Health, Bloomberg School of Public Health, Johns Hopkins University, USA

**Keywords:** Access, Healthcare, Immigrants, Japan

## Abstract

**Background:**

While Japan provides universal healthcare, immigrants may experience hampered access to healthcare. A comprehensive review of immigrant healthcare access is also lacking. This systematic review aims to examine barriers and facilitators of healthcare access among immigrants in Japan.

**Methods:**

We searched for literature published in English and Japanese until January 9, 2024. Studies were included if they assessed factors influencing any stage of immigrants’ healthcare access, such as perceiving needs, seeking, reaching, utilizing healthcare and the consequences of healthcare, as defined by the Levesque framework. We performed a thematic analysis to further identify categories (PROSPERO: CRD42023418554).

**Findings:**

After screening 2791 articles, we identified 67 studies (40 quantitative, 23 qualitative, 4 mixed methods) meeting eligibility criteria. Limited healthcare information led immigrants to seek alternative information sources and affected immigrants' perceived healthcare needs. Longer duration of stay improved access to healthcare information. Cultural and healthcare system differences affected healthcare seeking. Reaching and utilizing healthcare were hindered by heavy workloads, undocumented status, financial hardship, and limited insurance but were facilitated by support from family and friends. The healthcare system was often insufficient to support immigrants’ language and cultural needs leading to dissatisfaction and poor compliance.

**Interpretation:**

Findings highlight the critical importance of a multidimensional approach to support immigrants in Japan, ranging from improving healthcare information access to creating immigrant-friendly health systems. More research is needed on the healthcare access among vulnerable immigrants, such as undocumented and low-skilled labor immigrants and children.

**Funding:**

No specific funding source supported this study.

**Research in context**

**Evidence before this study**

Review on immigrant access to healthcare has not yet been conducted in high-income East Asian countries such as Japan or South Korea.

**Added value of this study**

After screening 2791 articles, we identified 67 studies (40 quantitative, 23 qualitative, 4 mixed methods) meeting eligibility criteria. We have identified barriers in all stages of healthcare access and facilitators in some stages of healthcare access according to Levesque et al. framework. There were few studies focusing on healthcare access for vulnerable groups, such as children, undocumented immigrants and low-skilled labor immigrants.

**Implications of all the available evidence**

A multidimensional approach is necessary to support immigrants—starting from providing accessible healthcare information and extending to immigrant-friendly health systems. More research is needed on the healthcare access among vulnerable immigrants, such as undocumented and low-skilled labor immigrants and children.

## Introduction

Migration has become an important social phenomenon with over 300 million immigrants globally.^1^ We define an immigrant as a person who moves to a country other than his or her usual residence and where the destination country becomes his or her new place of residence.^1^ Immigrants face different health risks throughout the migration journey, which vary by their immigration circumstances. Refugees are at risk of communicable diseases due to poor living conditions and mental health issues resulting from exposure to violence and trauma.^2^ Lack of official recognition by the host country may further limit their ability to seek healthcare.^2^ Labor immigrants, while generally healthier than the general population,^3^ can still be vulnerable due to the cumulative stress exposure that accompanies acculturation and the experiences of discrimination.^3^ Hence, the health status and healthcare needs of immigrants are multifaceted, influenced by their individual characteristics as well as the interaction of social, cultural and healthcare system of their countries of origin and their host countries.^4^

Immigrants tend to face more challenges in accessing healthcare than the host population.^4^ Here, according to Levesque's framework, we define “access to healthcare” as the opportunity for immigrants to reach and obtain appropriate healthcare services when needed.^5^ As outlined in the framework,^5^ access can be divided into stages, going from the identification of healthcare needs to the consequences of healthcare. Extensive literature reviews from high-income countries with significant immigrant populations, such as the United States,^3^ some European countries^2^ and Australia,^6^ show that even when legal access to healthcare is guaranteed, de facto access to healthcare can still be delayed due to differences in language, culture, knowledge and limited awareness of available services. However, such a review on immigrant access to healthcare has not yet been conducted in high-income East Asian countries such as Japan or South Korea. In these countries, healthcare systems have been less inclusive of immigrants while international immigration is rapidly increasing to address labor shortages caused by aging populations and a declining native work force.^7^ Understanding the barriers and facilitators to healthcare access for immigrants is crucial to establish healthcare policies that can improve access to healthcare for these populations.

Historically, Japan has had an ethnically homogenous population and restrictive migration policies, especially for low-skilled workers.^7^ Since the 1990s, due to the increase of the aging population and labor shortages, the Japanese government has amended the laws to allow the entrance of both high-skilled and low-skilled workers into the country.^7^ This has resulted in an increase in short-term low-skilled workers and language school students being allowed to work in non-regular, part-time jobs.^8^^,^^9^ As of December 2023, the percentage of immigrants in Japan was nearly 3%, corresponding to over 3 million immigrants.^10^ The largest population of immigrants in Japan were from China, Vietnam and South Korea.^10^ In terms of visa status, permanent residents accounted for 27% of the immigrant population, while 14% held technical intern visas (a temporary work visa of 3–5 years mainly for low skilled laborers), followed by 11% each for general office work visas (engineers, foreign language teachers, designers) and student visas.^10^ Regarding the immigrant age distribution in Japan, in 2023 over 50% of immigrants were in their 20s and 30s.^10^ The population of undocumented immigrants in Japan has remained quite low compared to other developed countries, i.e. less than 1% of the total population as of 2023 (with the majority of immigrants from Vietnam and Thailand).^11^ Since most immigrants in Japan are labor immigrants, the population influx is primarily driven by the push factor of economic instability in their home countries and pull factors of labor demand and the relatively high incentives offered in Japan.^7^

Japan has achieved universal health coverage since 1961, where almost all citizens are insured under employment-based or community-based insurance schemes.^12^ Until the Japanese government eliminated the need for citizenship in 1981, these insurance schemes did not cover immigrants.^7^ According to the law, it is mandatory for all residents, including immigrants, to enroll in health insurance, either through employment-based schemes for regular workers or community-based schemes for those without regular jobs.^13^ Except for persons over 70 years old with low incomes and children under age 3, the copayment is 30% while the premium rate varies, depending on income. Both insurance schemes need to be paid regularly by the individuals themselves or through their salaries.^13^ However, undocumented immigrants are not covered under the formal healthcare system and are excluded from both insurance schemes.^14^ Though Japan's health insurance targets universal coverage, many documented immigrants may also remain uninsured due to not understanding that insurance is mandatory.^15^ However, there is a lack of data on the exact percentages of uninsured immigrants throughout the country.

Apart from health insurance, the rapid increase of low skilled laborers in Japan has also posed significant challenges to achieving healthcare coverage as they often have notably poor access to healthcare because of language,^9^^,^^16^^,^^17^ cultural,^18^, ^19^, ^20^ and financial^21^^,^^22^ barriers, resulting in lower follow-up rates^22^ and higher mortality,^23^ compared to the native Japanese. Although interpreters might partly help to alleviate these challenges, there is also a scarcity of medical interpreters in Japan due to the labor shortage and the high cost of hiring them.^24^

To truly achieve the universal health coverage in Japan, poor access to healthcare for immigrants needs to be addressed. While there is some research on immigrants in Japan and their access to healthcare, most is focused on specific perspectives, for example, on a particular nationality of immigrants in a specific region (e.g.: Brazilians in Shiga prefecture).^25^ This narrow scope may overlook the broader background literature and the variations in healthcare access experienced by immigrants across Japan. Our systematic review aims to address this gap by using a mixed-methods approach, to comprehensively synthesize the literature on the barriers and facilitators on immigrants’ access to healthcare in Japan.

## Methods

### Literature search and selection criteria

We conducted this systematic review according to the Preferred Reporting Items for Systematic Reviews and Meta-Analyses (PRISMA) guidelines.^20^ We initially conducted a literature search for articles published until April 20, 2023, in the following databases: PubMed (MEDLINE), CINAHL, Web of Science using English search terms, and Igaku-chuo-zasshi (Ichushi), a Japanese biomedical literature database, using Japanese search terms. Additionally, a Google search was performed to capture the grey literature using both English and Japanese using recommended methodology for searching grey literature.^26^ Based on the Cochrane recommendations, we performed a follow up literature search for any updated literature between April 2023 and January 9, 2024.^27^ Search terms included “health service”, “healthcare”, “use”, “utilization”, “access”, “migrant”, “immigrant”, “refugee”, “foreign residents” and “Japan” which are terms that had been used in prior reviews on immigrant healthcare access^6^ or immigrants in Japan.^28^ Details of our search are described in Supplementary Methods 1. Studies were included if they: (1) targeted immigrants residing in Japan, (2) included Japanese nationals with or without comparison, (3) assessed barriers and facilitators influencing any stage of healthcare access either qualitatively or quantitatively or both, (4) published in either Japanese or English, and (5) presented original research data. As our review is on healthcare access, we excluded studies focused on immigrant health status in Japan but did not include information on access to healthcare. We also searched the reference lists of included studies for possible relevant titles. Rayyan^29^ and Mendeley^30^ web applications were used for reference management.

### Screening articles and data extraction

Two researchers (YPK, FMO) reviewed the study titles and abstracts independently, and if a study met the selection criteria, the full text was retrieved for further evaluation. Data were then extracted independently using a spreadsheet that included the study design, sample size, target population, data collection and analysis, theoretical background and results. Disagreements between the two researchers (which occurred in three instances) were resolved by discussion, and a third author (NN or TF) weighed in to reach consensus when necessary.

### Quality assessment

We used the 2018 version of the mixed methods appraisal tool (MMAT) developed by Hong et al. (2018)^31^ for assessment of study quality. This tool is specifically designed for mixed methods reviews (including components for qualitative studies, quantitative descriptive studies, and mixed-methods studies). The tools focus on five main areas, such as the appropriateness of the research methodology, data collection, data analysis, interpretation, and coherence of the entire study. The details of the appraisal tool are described in Supplementary Methods 2. Two independent researchers (YPK, FMO) rated the articles with “Yes (Y)”, “No (N)” and “Cannot tell (C)” for each criterion and discussed any disagreements. The overall quality of each study was then determined by the proportion of “Yes (Y)” scores received. Studies scoring fewer than three ‘Yes’ responses were classified as low quality, those with exactly three were considered moderate quality, and those with more than three were designated as high quality according to Hong et al., 2018.^31^

### Data transformation and analysis

As our research question was informed by both qualitative or quantitative methods, we followed the convergent integrated approach recommended by the Joanna Briggs Institute (JBI) Mixed Methods Review Methodology Group.^32^ We first codified the quantitative findings by converting them into textual descriptions (qualitative data), as this approach is less prone to error than quantifying the qualitative data. The converted textual data was then pooled with the qualitative data extracted from the qualitative studies and from the qualitative part of the mixed-methods studies. Thematic analysis was subsequently applied to the pooled data, following the six steps described by Braun and Clarke^33^: familiarization with data, generating initial codes, searching for themes and subthemes among the codes, reviewing themes, and creating theme names. Preliminary codes were aligned with the stages of the Levesque framework for access to healthcare, categorized as either barriers or facilitators and grouped into subthemes and main themes. We focused on the stages of the Levesque framework that represent the interaction between the accessibility of the Japanese healthcare system and the corresponding ability of immigrants to interact with it. These stages include the perception of needs and desire for care, healthcare seeking, healthcare reaching, healthcare utilization and the consequences of receiving healthcare.^5^ The final themes and subthemes were discussed by at least three researchers until agreement was reached.

### Role of the funding source

There was no funding source that supported this study. Authors were not precluded from accessing data in the study, and they accept responsibility to submit for publication.

## Results

**Fig. 1:**
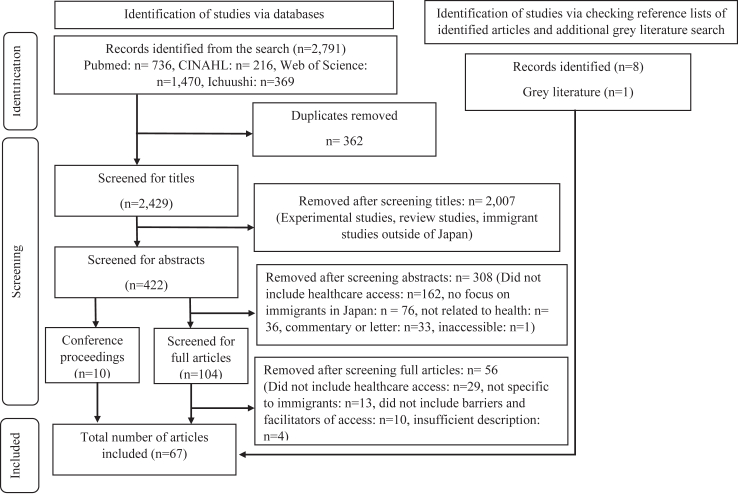
Study selection.

We retrieved a total of 2791 potentially relevant citations from the search, from which 362 duplicates were removed. After screening titles and abstracts, the full text of 104 articles was examined. Finally, after consideration of ten conference proceedings, eight additional articles identified through checking reference lists and 1 article found in the grey literature search, 67 articles met the eligible criteria and were included (Fig. 1).

**Fig. 2:**
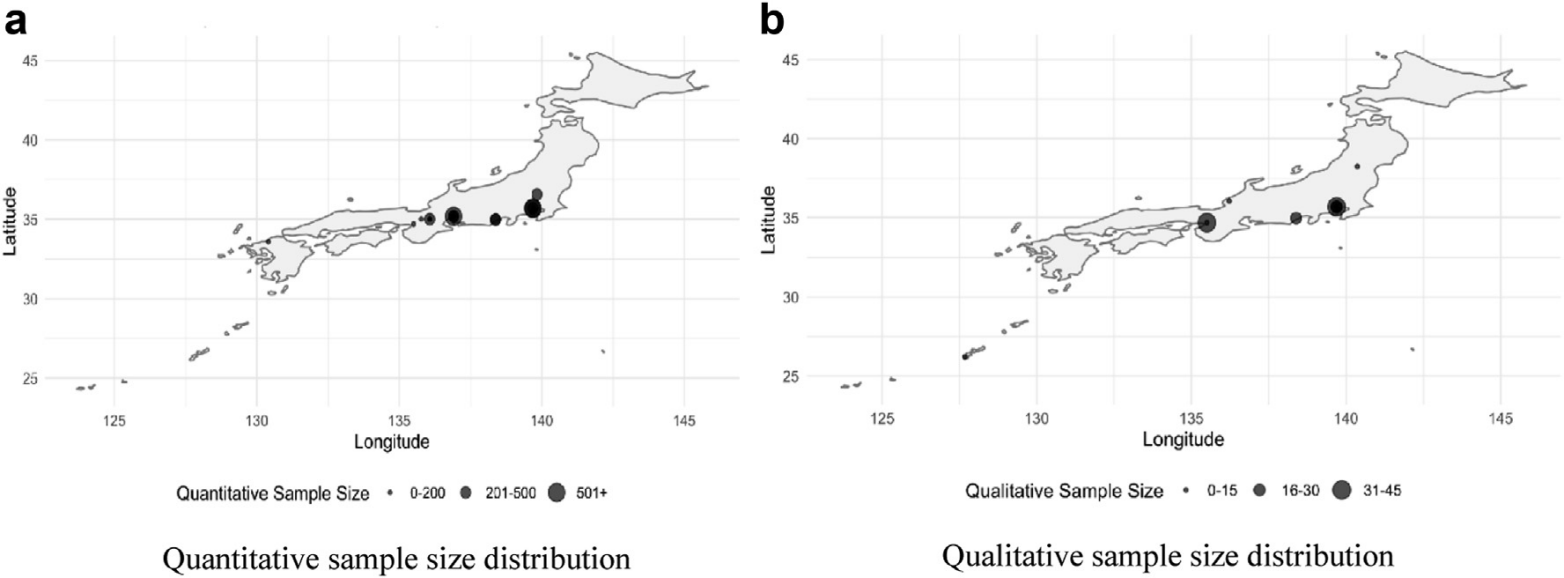
These maps illustrate the sample sizes of the included studies with specified prefecture (n = 38). Figure 2(a) represents samples from quantitative (n = 26) and the quantitative part of mixed-methods studies (n = 2) while Figure 2(b) represents samples from qualitative studies (n = 10) and the qualitative part of mixed-methods studies. Within each figure, the sizes are proportionate to the sample sizes. For simplifications, five studies that specified the region as Greater Tokyo Area or Kanto region are represented by Tokyo prefecture and two studies which specified as Kansai region are represented by Osaka prefecture.

Table 1 displays the characteristics of the included studies: 40 used quantitative methods, 23 used qualitative methods, and 4 used mixed methods. Most quantitative studies involved fewer than 100 immigrants (n = 17, 42.5%). Quantitative data were primarily collected through questionnaires (n = 30, 75%), and qualitative data through interviews (14, 60.9%). Among all included studies, four studies compared immigrants' healthcare access to the Japanese population (n = 4, 6.0%) and two included healthcare providers' opinions (n = 2, 3.0%). Over one-third of the studies were published between 2020 and 2023 (n = 24, 35.8%). In terms of the healthcare stages outlined in Levesque's framework,^5^ most studies focused on the perception of needs and desire for care (n = 26, 38.8%) and healthcare consequences (n = 37, 55.2%). Nearly half of the studies did not specify the participants' country of origin, whereas 25.4% of the studies included immigrants from Latin America (n = 17), followed by Southeast Asian (n = 15, 22.4%), East Asian (n = 9, 13.4%) and South Asian (n = 7, 10.4%) immigrants. Most studies did not mention the visa status of the participants (n = 54, 80.6%). Geographically, studies were concentrated in the Kanto (n = 19, 28.4%), Kansai (n = 9, 13.4%) and Chubu (n = 7, 10.4%) regions, which have the highest immigrant populations.^10^ Fig. 2 illustrates the sample sizes of studies with the prefecture specified (n = 38). Supplementary Tables S1, S2 and S3 display the details of each study.

**Table 1:** Characteristics of the studies included (N = 67).

Number of studies	Total (n, %)	Quantitative (40, 59.7%)	Qualitative (23, 34.3%)	Mixed methods (4, 6.0%)
**Type of study**				
Original articles	56, 83.6%	34, 85.0%	18, 78.2%	4, 100%
Grey literature including conference proceedings	11, 16.4%	6, 15%	5, 21.7%	0
**Quantitative sample size**^a^				
More than 1000	–	2, 5.0%	–	0
500–1000	–	6, 15.0%	–	2, 50%
100–499	–	15, 37.5%	–	2, 50%
<100	–	17, 42.5%	–	0
**Qualitative sample size**^a^				
>20	–	–	7, 30.4%	2, 50%
10–19	–	–	9, 39.1%	1, 25%
<10	–	–	7, 30.4%	1, 25%
**Quantitative source of data**				
Questionnaire survey	–	30, 75.0%	–	4, 100%
Review of records	–	9, 22.5%	–	0
Both primary data and review of records	–	1, 2.5%	–	0
**Qualitative source of data**				
Interview	–	–	14, 60.9%	2, 50%
Focus group discussion	–	–	4, 17.4%	2, 50%
Interview and participant observation	–	–	4, 17.4%	0
Interview and focus group discussion	–	–	1, 4.3%	0
**Include comparison with the Japanese population**				
Present	4, 6.0%	4, 10	0	0
Absent	63, 94.0%	36, 90%	23, 100%	4, 100%
**Include healthcare providers**				
Present	2, 3.0%	1, 2.5%	1, 4.3%	0
Absent	65, 97.0%	39, 97.5%	22, 95.7%	4, 100%
**Year of publication**				
2020–2023	24, 35.8%	11, 27.5%	9, 39.1%	4, 100%
2015–2019	15, 22.4%	9, 22.5%	6, 26.1%	0
2010–2014	9, 13.4%	5, 12.5%	4, 17.4%	0
2005–2009	5, 7.5%	3, 7.5%	2, 8.7%	0
2000–2004	8, 11.9%	7, 17.5%	1, 4.3%	0
1995–1999	3, 4.5%	3, 7.5%	0	0
1992–1994	3, 4.5%	2, 5.0%	1, 4.3%	0
**Stage of access to healthcare**^b^				
Perception of needs and desire for care	26, 38.8%	15, 37.5%	8, 34.8%	3, 75%
Healthcare seeking	15, 22.4%	3, 7.5%	9, 39.1%	3, 75%
Healthcare reaching	15, 22.4%	8, 20.0%	5, 21.7%	2, 50%
Healthcare utilization	20, 29.9%	17, 42.5%	1, 4.3%	2, 50%
Healthcare consequences	37, 55.2%	20, 50.0%	15, 65.2%	2, 50%
**Immigrants' country of origin**^b^				
Not specified	26, 38.8%	17, 42.5%	9, 39.1%	0
East Asian	9, 13.4%	5, 12.5%	3, 13.0%	1, 25%
Southeast Asian	15, 22.4%	6, 15.0%	6, 26.1%	3, 75%
South Asian	7, 10.4%	4, 10.0%	1, 4.3%	2, 50%
Latin American	17, 25.4%	12, 30.0%	5, 21.7%	0
European, Australia, US	1, 1.5%	1, 2.5%	0	0
**Type of visa**^b^				
Not specified	54, 80.6%	35, 87.5%	16, 69.6%	3, 75%
Refugee and undocumented	1, 1.5%	1, 2.5%	0	1, 25%
Technical Intern^c^	3, 4.5%	0	3, 13.0%	1, 25%
Students	6, 9.0%	3, 7.5%	3, 13.0%	1, 25%
Highly skilled visa	1, 1.5%	0	1, 4.3%	0
Spouse visa	1, 1.5%	0	1, 4.3%	0
Long-term/Permanent resident	3, 4.5%	1, 2.5%	2, 8.7%	0
**Region of study**				
Nationwide	13, 19.4%	10, 25.0%	1, 4.3%	2, 50%
Tohoku	1, 1.5%	0	1, 4.3%	0
Kanto	19, 28.4%	13, 32.5%	4, 17.4%	2, 50%
Kansai	9, 13.4%	6, 15.0%	3, 13.0%	0
Chubu	7, 10.4%	6, 15.0%	1, 4.3%	0
Kyushu	1, 1.5%	1, 2.5%	0	0
Okinawa	1, 1.5%	0	1, 4.3%	0
Not specified	16, 23.9%	4, 10.0%	12, 52.2%	0

a Sample size represents only immigrant participants.

b Percentages might not add up to 100 due to some studies covering multiple categories (i.e. more than one country of origin, type of visas or stage of access to healthcare).

c Technical intern: Temporary work visa of 3–5 years (mainly for low skilled laborers).

**Fig. 3:**
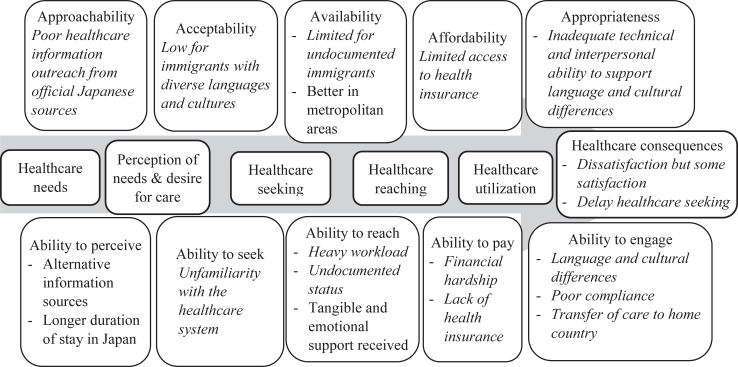
Barriers and facilitators that immigrants in Japan face to access healthcare according to the Levesque conceptual framework of healthcare access. (Barriers are focused with italic text).

Barriers and facilitators to healthcare access among immigrants in Japan are presented in Table 2 and illustrated in Fig. 3 under the following stages of the Levesque framework for healthcare access^5^: (1) perception of needs and desire for care: receiving healthcare information, (2) healthcare seeking: navigating through health system and cultural differences, (3) healthcare reaching (4) healthcare utilization, and (5) healthcare consequences for immigrants.

(1) Perception of needs and desire for care: Receiving healthcare information

**Table 2:** Barriers and facilitators that immigrants in Japan faced in accessing healthcare.

Stages of healthcare access according to Levesque's framework of healthcare access^5^	Barriers	Facilitators
Perception of needs and desire for care: Receiving healthcare information	Poor healthcare information outreach from official Japanese sources^9^^,^^16^^,^^21^^,^^34^, ^35^, ^36^, ^37^, ^38^, ^39^, ^40^, ^41^, ^42^, ^43^, ^44^	Alternative information sources from immigrant communities, NGOs, and from home country^21^^,^^39^^,^^41^^,^^45^, ^46^, ^47^, ^48^, ^49^, ^50^, ^51^, ^52^, ^53^, ^54^, ^55^
		Longer duration of stay in Japan^41^^,^^42^^,^^56^
Healthcare seeking: Navigating through health system and cultural differences	Unfamiliarity with the Japanese healthcare system^16^^,^^21^^,^^57^, ^58^, ^59^, ^60^, ^61^	
	Low acceptability of Japanese healthcare for immigrants with diverse languages and cultures^48^^,^^49^^,^^51^^,^^52^^,^^56^^,^^57^^,^^60^, ^61^, ^62^, ^63^	
Healthcare reaching	Lack of time due to immigrants' heavy workloads^8^^,^^61^^,^^64^	Tangible and emotional support from family, friends, neighbors and employers^8^^,^^16^^,^^47^^,^^65^, ^66^, ^67^, ^68^, ^69^, ^70^
	Undocumented status^21^^,^^52^^,^^71^	Favorable place of residence: metropolitan areas^72^
Healthcare utilization	Financial hardship^37^^,^^48^^,^^52^^,^^64^^,^^73^^,^^74^	
	Limited access to health insurance^14^^,^^15^^,^^53^^,^^54^^,^^57^^,^^67^^,^^71^, ^72^, ^73^^,^^75^, ^76^, ^77^, ^78^, ^79^	
Healthcare consequences for immigrants	Inadequate technical and interpersonal ability to support language^16^^,^^17^^,^^19^^,^^21^^,^^35^^,^^37^^,^^46^^,^^53^^,^^54^^,^^58^^,^^60^^,^^62^^,^^66^^,^^70^^,^^75^^,^^80^, ^81^, ^82^, ^83^, ^84^, ^85^ and cultural^21^^,^^40^^,^^58^^,^^60^, ^61^, ^62^^,^^80^^,^^81^^,^^83^^,^^86^, ^87^, ^88^, ^89^ differences	
	Dissatisfaction^40^^,^^59^^,^^61^ and delay in seeking healthcare for future illnesses^43^^,^^45^^,^^90^	Satisfaction with healthcare^66^^,^^80^^,^^83^^,^^84^^,^^87^^,^^88^
	Poor compliance^20^^,^^62^^,^^67^^,^^76^^,^^77^^,^^79^^,^^91^	
	Transfer of care to home country during treatment^72^^,^^76^

Most studies mentioned the challenges faced by immigrants in receiving information for seeking appropriate healthcare. Limited access to official healthcare information was provided to immigrants by the Japanese government.^16^^,^^21^^,^^34^, ^35^, ^36^, ^37^ Since the majority of information was in Japanese, immigrants with poor Japanese proficiency were unaware of the availability of health services,^38^, ^39^, ^40^, ^41^, ^42^, ^43^ including some services provided free of charges.^9^^,^^44^^,^^45^

As a result, immigrants relied on alternative information sources such as family, friends, and immigrant communities^46^, ^47^, ^48^, ^49^, ^50^ which sometimes included doctors and nurses from their countries of origin.^21^ Many also relied on the healthcare information from their home countries via the internet and social media.^21^^,^^39^^,^^41^^,^^51^^,^^52^^,^^57^ Concerning healthcare services requiring privacy concern such as HIV treatment, information from non-governmental organizations (NGO) was helpful.^52^, ^53^, ^54^ Immigrants’ having access to information from official sources such as public health centers or municipalities resulted in better access to healthcare^45^^,^^55^ compared to reliance on information from alternative sources.^41^

Duration of stay in Japan was linked with immigrants’ ability to receive healthcare information. The longer the immigrants stayed in Japan, they demonstrated better health literacy,^42^ a greater ability to seek information^41^^,^^56^ and were more likely to visit doctor when needed.^45^

(2) Healthcare seeking: Navigating through health system and cultural differences

Many studies described the difficulties faced by immigrants in becoming familiar with Japanese healthcare system.^21^^,^^58^ Some difficulties were due to differences between immigrants’ home countries and Japan in the availability of healthcare options,^16^^,^^59^ prescription procedures and payment systems.^60^ These difficulties were particularly emphasized at times when healthcare needs substantially increased, for example around the time of delivery and caring for children.^57^^,^^61^

While seeking healthcare, immigrants preferred services that met their linguistic and cultural needs, but Japanese healthcare facilities fell short of meeting these requirements.^48^^,^^49^^,^^57^^,^^61^^,^^62^ Immigrants from English-speaking countries were able to find doctors who could communicate in English.^49^^,^^51^^,^^63^ Those from Latin America (with a long history of migration to Japan)^7^ were also able to find interpreters^56^ in tertiary hospitals equipped with interpretation systems to accommodate foreign patients.^60^ However, for immigrants from other non-English speaking countries, medical interpretation that was needed to obtain appropriate healthcare became challenging.^52^

(3) Healthcare reaching

Immigrants with heavy workloads, for example labor intensive jobs (e.g. factory workers and technical intern trainees)^8^^,^^64^ as well as working mothers^61^ faced difficulties prioritizing their health and therefore accessing healthcare. In many cases, support from their employer or supervising organizations was required for them to obtain healthcare.^8^ Documentation status also significantly influenced access to healthcare, as undocumented immigrants were excluded from formal healthcare services.^21^^,^^52^^,^^71^ However, in metropolitan areas such as Tokyo, undocumented immigrants were more easily able to obtain healthcare for infectious diseases like tuberculosis than in rural areas.^72^

Emotional support from family, friends, and neighbors facilitated reaching healthcare among immigrants,^65^, ^66^, ^67^, ^68^, ^69^^,^^92^ as did tangible support such as being accompanied by an interpreter.^16^^,^^47^^,^^65^^,^^68^ International students who were less likely to be accompanied by their families in Japan had low social support for reaching healthcare.^70^

(4) Healthcare utilization

Financial hardship posed significant barriers for immigrants to utilize healthcare, especially for students or technical interns^52^^,^^73^ as well as for those with low incomes^48^^,^^64^^,^^74^ or without health insurance.^67^^,^^72^^,^^73^^,^^75^, ^76^, ^77^ High out-of-pocket payments without insurance caused some immigrants to stop medical treatment in the middle as they were not able to cover the costs.^37^^,^^67^^,^^78^ However, health insurance systems in Japan have not covered undocumented immigrants.^14^^,^^71^ Even among documented Latin American immigrants, health insurance enrollment was low, due to limited knowledge and high premiums.^15^^,^^78^^,^^79^ However, immigrants with chronic health conditions, such as HIV infection, tended to actively seek health insurance.^53^^,^^54^

(5) Healthcare consequences for immigrants

The language barrier between immigrants and healthcare providers was a major issue for accessing healthcare, affecting immigrants from various backgrounds^16^^,^^17^^,^^19^^,^^21^^,^^46^^,^^60^^,^^62^^,^^75^^,^^80^, ^81^, ^82^ including East Asian immigrants, whose languages may be perceived to be similar to Japanese.^35^^,^^83^ Several studies highlighted the need for interpreters, and that the use of professional interpreters improved healthcare access for immigrants.^35^^,^^70^^,^^84^ However, professional interpreters were scarce and only available during limited hours^37^^,^^58^^,^^66^ and many immigrants were forced to find interpreters on their own.^53^^,^^54^ Strategies were used to address this barrier, such as the use of non-professional interpreters^66^ and translation software.^58^ However, these approaches are limited, as their reliability and accuracy may be inadequate.^58^^,^^85^

Immigrants were also concerned with lack of cultural competency by healthcare providers.^21^^,^^62^^,^^80^^,^^86^ Cultural differences were more pronounced in healthcare related to maternal and child health, where cultural expectations play a significant role.^61^^,^^83^^,^^87^^,^^88^ In addition, Japanese dietary instructions provided by healthcare providers during hospitalization or diabetic treatments were difficult to follow for immigrants with different dietary habits.^81^^,^^89^ Particular concerns of immigrants were related to privacy, especially for gynecological examinations.^60^ Some immigrants also expressed willingness to discuss more with doctors to clarify their health conditions, however, in Japan, the communication with doctors tends to be one-way, i.e. from doctors to patients.^58^^,^^60^^,^^61^ Some Latin American and Nepalese women also expressed concerns that the dose of the drugs prescribed to them was not sufficient to relieve their symptoms.^21^^,^^40^

Many immigrants expressed dissatisfaction because the Japanese healthcare system did not accommodate their diverse needs.^40^^,^^59^^,^^61^ This further delayed access to healthcare,^43^^,^^45^^,^^90^ leading immigrants to refrain from visiting hospitals or clinics despite ill health. Even upon reaching healthcare facilities, many were unable to build trust with healthcare providers,^20^^,^^62^ leading to poor compliance with treatment, including termination of treatment^67^^,^^76^^,^^77^^,^^91^ and frequent self-medication.^79^ When symptoms became severe, many immigrants sought treatment in their home countries.^72^^,^^76^ However, some immigrants, particularly from Latin America,^66^^,^^84^ English-speaking countries^87^ and China,^83^^,^^88^ expressed satisfaction with access to health care.^80^

### Study quality

The MMAT^31^ was used to assess the quality of the included papers. Over 85% of studies received more than three “yes” ratings, out of five criteria representing moderate to high quality. The results of the quality scores are presented for quantitative studies in Supplementary Table S2, for qualitative studies in Supplementary Table S3, and for mixed-methods studies in Supplementary Table S4.

## Discussion

To our knowledge, this is the first systematic review to examine barriers and facilitators to healthcare access of immigrants in Japan, including an analysis of all stages of access and literature in both English and Japanese. We have identified barriers in all stages of healthcare access and facilitators in some stages of healthcare access according to Levesque et al. framework.^5^ In the first stage of healthcare access, the approachability of Japanese healthcare services was limited,^16^^,^^21^^,^^34^, ^35^, ^36^, ^37^ as official government information was predominantly in Japanese,^38^, ^39^, ^40^, ^41^, ^42^, ^43^ causing immigrants to rely on alternative sources from their communities and home countries, usually through social media.^21^^,^^39^^,^^41^^,^^51^^,^^52^^,^^57^ These sources differed from those of the predominantly elderly Japanese population, who relied primarily on mass media like television and digital platforms such as news websites.^93^ In contrast, immigrants in Japan are mostly young (15–34 years old)^10^ tend to depend on social media, preferring sources which they have used previously, such as those from their immigrant communities and home countries.^41^ This is similar to immigrants in Australia^94^ who preferred information from sources with which they had direct connections and which they considered reliable.

While immigrants faced difficulties in seeking healthcare due to unfamiliarity with the Japanese healthcare system, the system had low acceptability for immigrants. The majority of clinics and hospitals in Japan were not adequately prepared to accommodate the diverse language and cultural needs (we defined culture as the beliefs and values related to receiving healthcare)^5^ of immigrants,^48^^,^^49^^,^^57^^,^^61^^,^^62^ with the exception of tertiary hospitals with foreigner-friendly systems.^60^ This was reflected in a report by the Tokyo metropolitan government, which noted that foreigners readily used tertiary hospitals, which incurred extra charges due to the lack of referrals from primary care physicians, though it was unclear whether these foreigners were immigrants or foreign tourists.^95^

Reaching healthcare was particularly challenging for undocumented immigrants and those with heavy workloads, but receiving social support improved their ability to reach healthcare. Predictably, undocumented immigrants had limited access to healthcare,^21^^,^^52^^,^^71^ though some metropolitan municipalities provided support for infectious diseases due to public health concerns.^72^ Unlike in some Asian and European countries where the government provides full healthcare access to undocumented immigrants,^2^^,^^96^ undocumented immigrants in East Asian countries such as Japan and Korea still face challenges in accessing proper healthcare, because of the unfavorable political and public stance toward them.^71^ While social support is beneficial for immigrants to reach healthcare, migration often results in a breakdown of social networks and pre-existing support, especially for international students^70^ and labor immigrants who are not allowed to bring their families.^8^

Limited access for immigrants to health insurance was a key determinant of healthcare utilization.^67^^,^^72^^,^^73^^,^^75^, ^76^, ^77^ Japan has achieved universal health coverage, with less than 5% of Japanese citizens uninsured.^12^ In contrast, the proportion of uninsured immigrants was notably high,^65^ up to 35% among Nepalese immigrants^75^ and was even notably high among documented immigrants.^15^ High uninsurance among documented immigrants appears to be specific to Japan, and contrasts with other countries with universal health coverage where not having insurance was a main concern for only undocumented immigrants.^2^ When immigrants migrate to Japan, they are automatically enrolled in the insurance system^13^; however, to maintain their status, they need to pay insurance premiums themselves on a regular basis especially when under community-based insurance plans.^15^ If they are unaware of this due to language barriers or to differences between the Japanese health insurance system and that in their home countries, they will lose insurance coverage and are faced large premium back payments to regain their insured status.^15^ The lack of ability to make these back payments has led to low insurance rates, regardless of the documentation status.^15^

Our review found that the healthcare system in Japan was inappropriate for immigrants due to inadequate language^16^^,^^17^^,^^19^^,^^21^^,^^46^^,^^60^^,^^62^^,^^75^^,^^80^, ^81^, ^82^ and cultural^21^^,^^40^^,^^61^^,^^62^^,^^80^^,^^83^^,^^86^, ^87^, ^88^ support for immigrants. This resulted in dissatisfaction with the health system,^40^^,^^59^^,^^61^ delays in seeking care,^43^^,^^45^^,^^90^ and poor compliance^67^^,^^76^^,^^77^^,^^91^ among immigrants. While some measures have been taken to reduce language barriers, these also have had limitations.^37^^,^^58^^,^^66^^,^^85^ A review of translation devices for immigrants suggested that an integrated electronic interpretation system that included easy-to-understand pictorial guides, the option to call a professional interpreter during emergencies, cultural adaptation and a back translation function would be the most acceptable for immigrants.^97^

This review has some limitations. First, all studies were single time point descriptive studies where the policies for healthcare access during migration differed according to the time of migration. Second, the sample characteristics, methodology, and outcomes varied across studies. To analyze publication bias using a funnel plot or forest plot, the outcomes need to be consistent. Therefore, we could not statistically evaluate the presence of publication bias and instead conducted a narrative meta-synthesis. Third, despite healthcare being the interface between providers and recipients, we excluded articles that solely included healthcare providers' perspectives, as it was unclear whether their perspectives applied specifically to immigrants or included temporary residents.

Despite these limitations, we included 67 diverse studies from 1992 to 2023 and applied minimal restrictions on publication date, language, methodology, and type of publication, ensuring a comprehensive review of immigrants' access to healthcare. Additionally, we converted the quantitative data to qualitative data following recommendations from the Mixed Methods Review Methodology Group^32^ to minimize potential errors. Before the 1990s, immigrants in Japan faced significant discrimination and were referred to as ‘aliens.’ Citizenship was required to access social welfare, including health insurance.^7^ After our study period, Japan will become more internationalized, particularly with an influx of low-skilled labor immigrants from developing countries in Asia, and new visa statuses expanding their job opportunities.^10^

To ensure better healthcare access for immigrants in the future, we have identified the following research gaps. There were few studies focusing on healthcare access for vulnerable groups, such as children, undocumented immigrants and low-skilled labor immigrants. Healthcare access for international students, those attending Japanese language schools, vocational schools and universities should be differentiated, as they often have different socio-economic dynamics.^9^^,^^80^ Moreover, only a few quantitative studies,^48^^,^^56^^,^^63^ some of which are of low quality,^48^^,^^63^ were conducted on healthcare seeking, despite qualitative research indicating that immigrants face difficulties in seeking healthcare services. There is a need to quantify immigrants' evaluations of the acceptability of the Japanese healthcare system, e.g. how the system considers immigrants’ treatment needs, taking into account the significant variation in their background characteristics. Studies on healthcare utilization that included financial factors were predominantly quantitative, indicating a need to qualitatively explore immigrants' perspectives regarding the health insurance system and healthcare costs in Japan. Regarding the healthcare consequences, some studies indicated that immigrants were satisfied with the Japanese healthcare system,^60^^,^^66^^,^^83^^,^^84^^,^^87^^,^^88^ though half of these studies were of low quality and over ten years old.^60^^,^^66^^,^^84^ There is a need for high-quality research to identify the specific factors that contribute to satisfaction.

We would like to highlight several implications of this body of diverse research. For immigrants to have better access to official healthcare information, government bodies should work in collaboration with immigrant communities to ensure that the information is linguistically and culturally tailored to the immigrant population. Future research should explore whether influential leaders from immigrant communities and the use of social media can improve healthcare access for this population. It is necessary to develop an immigrant friendly health system, including ensuring access to health insurance for immigrants and extending healthcare coverage to those who are undocumented. Government and supporting organizations should support immigrants from non-English-speaking countries by providing reliable and affordable translation systems with cultural adaptations. Further promotion of cultural awareness among healthcare providers should also be encouraged. Community-based initiatives in collaboration with NGOs (e.g. language classes and cultural exchange programs) to improve immigrant social networks are also necessary. Finally, our review suggested further research is needed on immigrants’ access to healthcare throughout the stages of migration.

In conclusion, a multidimensional approach is necessary to support immigrants—starting from providing accessible healthcare information and extending to immigrant-friendly health systems. These systems should be equipped to ensure access to health insurance, reliable and affordable language interpretation services, and culturally appropriate healthcare. Additionally, social networks that support immigrants’ access to healthcare should be encouraged.

## Contributors

YPK formulated the research question, designed the study, performed the literature search, analyzed the data, interpreted the findings and prepared the original manuscript. FMO contributed to the design of the study, analysis of the data and interpretation of the findings. NN contributed to the analysis of the data, interpretation of the findings and reviewed the article. PJS reviewed and edited the article. TF contributed to the analysis of the data, interpretation of the findings and reviewed the article.

## Data sharing statement

Since this study was a systematic review and meta-synthesis of published studies and grey literature, all data used are publicly available in the databases specified in the methods section.

## Editor note

The Lancet Group takes a neutral position with respect to territorial claims in published maps and institutional affiliations.

## Declaration of interests

In addition to her contributions to the research in this article, Dr. Surkan also has taught a short course on qualitative research methods at the Institute of Science Tokyo (formerly known as Tokyo Medical and Dental University). This arrangement has been reviewed and approved by the Johns Hopkins University in accordance with its conflict of interest policies.

## Appendix A Supplementary data

Supplementary Tables

Suppl_protocol
