# Supplementary material for: Barriers and facilitators for healthcare access among immigrants in Japan: a mixed methods systematic review and meta-synthesis
Source: Lancet Reg Health West Pac. 2025 Jan 10;54:101276. doi: 10.1016/j.lanwpc.2024.101276 (PMC11774800; doi:10.1016/j.lanwpc.2024.101276)
Supplement: Supplementary Tables [file mmc1.docx]

**Supplementary Methods 1**

**Search terms**

**PubMed**

("Health service*"[Text Word] OR "health care"[Text Word] OR "Healthcare"[Text Word] OR "Specialist*"[Text Word] OR "Hospitalization*"[Text Word] OR “Hospitalization*”[MeSH Terms] OR "Mental"[Text Word] OR "Vaccin*"[Text Word] OR “Vaccination”[MeSH Terms] OR "health check-up*"[Text Word] OR "primary service*"[Text Word] OR "dental"[Text Word] OR “health services accessibility"[Text Word] OR “health services accessibility”[Text Word] OR "health services accessibility"[MeSH Terms] OR “health”[Text Word] OR "health"[MeSH Terms] OR "use"[Text Word] OR "usage"[Text Word] OR "utilize"[Text Word] OR "utilization"[Text Word] OR "access"[Text Word])

AND

("Migrant*"[Text Word] OR "Immigrant*"[Text Word] OR “Refugee*” [MeSH Terms] OR "Refugee*"[Text Word] OR "Migration"[Text Word] OR "Foreigner*"[Text Word] OR "Foreign resident*"[Text Word] OR "International student*"[Text Word])

AND

("Japan"[Text Word] OR "Japanese"[Text Word])

**CINAHL**

(MH(“Health service+”) OR MH(“Health service accessibility”) OR MH(“healthcare”) OR "Health service*" OR "health care" OR "Healthcare" OR MH(“specialized healthcare”) OR "Specialized health service*"OR MH(“Specialist”) OR "Specialist*" OR MH(“Hospitalization”) OR "Hospitalization" OR MH(“health check-ups”) OR "health check-up*" OR "primary service*" OR MH(“dental”) OR "dental" OR MH(“vaccin*”) OR "vaccin*")

AND

(MH(“Transient”) OR "Transient" OR MH(“Migrant+”) OR "Migrant*" OR MH(“Refugee+”) OR "Refugee*" OR MH(“Migration background”) OR "Migration background" OR "Immigrant background" OR MH(“Immigrant+”) OR "Immigrant*" OR MH(“Foreigner+”) OR "Foreigner*" OR MH(“Foreign resident”) OR "Foreign resident*" OR MH(“International student+”) OR "International student*" OR MH ("use") OR MH ("usage") OR MH ("utilize") OR MH ("utilization") OR MH ("access")

AND

TX (Japan or Japanese))

**Web of Science**

("Health service*" OR "health care" OR "Healthcare" OR "Specialist" OR "Hospitalization" OR "Mental" OR "Vaccination" OR "health check-up*" OR "primary service" OR "dental" OR "health services accessibility" OR "health" OR "use" OR "usage" OR "utilize" OR "utilization" OR "access")

AND

("Migrant*" OR "Immigrant*" OR "Refugee" OR "Migration"[Text Word] OR "Ethnic minority" OR "Foreigner*" OR "Foreign resident*" OR "International student*")

AND

("Japan" OR "Japanese")

**Ichushi (Japanese search terms)**

((保健医療サービス/TH) or (保健医療サービス/AL) or (保健医療サービス利用可能性/TH) or (保健医療サービス利用可能性/AL) or (保健医療政策/TH) or (保健医療政策/AL) or (病院診療科/TH) or (病院診療科/AL) or (精神保健サービス/TH) or (精神保健サービス/AL) or (予防的保健医療サービス/TH) or (予防的保健医療サービス/AL) or (歯科医療サービス/TH) or (歯科医療サービス/AL) or (保健医療の品質, アクセス, 評価/TH) or (使用/AL) or (アクセス/AL))

and

((外国人/TH) or (外国人/AL) or (国外移住/TH) or (国外移住/AL) or (留学生/AL) or (避難者/TH) or (避難者/AL) or (在日コリアン/TH) or (不法滞在者/TH) or (不法滞在者/AL))

and

((在日/AL) or (日本在住/AL) or (在留/AL))

**Grey Literature Search at Google**

**English search terms**

("Health service*" OR "health care" OR "Specialist*" OR "Hospitalization*" OR "Mental" OR "Vaccin*" OR "health check-up*" OR "primary service*" OR "dental" OR “health services accessibility” OR "health" OR "use" OR "usage" OR "utilize" OR "utilization" OR "access" AND ("Migrant*" OR "Immigrant*" OR “Refugee* OR "Migration" OR "Foreigner*" OR "International student*") AND ("Japan" OR "Japanese"):pdf

**Japanese search terms**

("保健医療サービス *" OR "病院診療科*" OR "精神" OR "予防*" OR "予防的保健医療サービス*" OR "プライマリーサービス*" OR "歯科医療" OR ""健康 OR "使用" OR "アクセス" AND ("外国人" OR "国外移住" OR "留学生" OR "避難者" OR "在日コリアン" OR "不法滞在者") AND ("在日" OR "日本在住"OR"在留"):pdf

Supplementary Methods 2

Mixed Method Appraisal Tool (MMAT)

1. For quantitative descriptive studies

1.1. Is the sampling strategy relevant to addressing the research question?

1.2. Is the sample representative of the target population?

1.3. Are the measurements appropriate?

1.4. Is the risk of nonresponse bias low?

1.5. Is statistical analysis appropriate to answer the research question?

2. For qualitative studies

2.1. Is the qualitative approach appropriate to answer the research question?

2.2. Are the qualitative data collection methods adequate to address the research question?

2.3. Are the findings adequately derived from the data?

2.4. Is the interpretation of results sufficiently substantiated by data?

2.5. Is there coherence between qualitative data sources, collection, analysis and interpretation?

3. Mixed-methods

3.1. Is there an adequate rationale for using a mixed methods design to address the research question?

3.2. Are the different components of the study effectively integrated to answer the research question?

3.3. Are the outputs of the integration of qualitative and quantitative components adequately interpreted?

3.4. Are divergences and inconsistencies between quantitative and qualitative results adequately addressed?

3.5. Do the different components of the study adhere to the quality criteria of each tradition of the methods involved?

| Supplementary Table 1: Characteristics of quantitative studies (N=40) | | | | | | |
| --- | --- | --- | --- | --- | --- | --- |
| First author and year of publication | Study population (Sample size, country of origin, type of visa (if applicable) and the location of study) | Objectives | Method of data collection | Stage of access to healthcare | Main results |  |
| Teng et al., 2023^1^ | 1455 immigrants from 48 countries residing across Japan | To explore COVID-19 vaccine intention and  acceptance and the association between integration and vaccine hesitancy among immigrants in Japan. | Internet survey | Healthcare reaching | Facilitator: Language and financial difficulties were not associated with vaccine acceptance. Highly social-integrated immigrants were less likely to report vaccine hesitancy (OR= 0.18). |  |
| Teng et al., 2023^2^ | 3690 participants (1485 foreign born immigrants, 501 Japan born immigrants and 1704 Japanese) residing across Japan. Immigrants were rooted mainly from East Asian countries. | To explore the differences in COVID-19 vaccination readiness and its association with vaccine acceptance among foreign-born immigrants, Japan-born immigrants, and Japanese. | Internet survey. | Perception of needs and desire for care | Facilitator: Immigrant participants reported higher general vaccination readiness and acceptability for vaccination against COVID-19 than the Japanese participants but had lower vaccination coverage. Confidence on vaccine and collective responsibility were low among immigrants. |  |
| Iglesias Rodriguez et al., 2023^3^ | 390 adult immigrants from Latin American countries residing across Japan. | To analyze the current situation of Chaga disease in Japan and identify possible barriers to seeking care. | Face-to-face survey at specified booths. | Perception of needs and desire for care, healthcare consequences | Barrier: Being female (OR=2.3), limited Japanese skills (OR=2.16), and dissatisfaction about the Japanese healthcare system (OR=10.06) were associated with not seeing a doctor when needed.  Facilitator: Length of stay in Japan>10 years (OR=0.31), receiving information from official sources (OR=0.13) were the facilitators. |  |
| Supakul et al., 2023^4^ | 67 Thai immigrants with short-term, long term and unspecified visas residing in Tokyo, Chiba and Ibaraki prefectures. | To access factors associated with health insurance among vulnerable Thai immigrants. | Face-to-face survey at public religious events. | Healthcare utilization | Barrier: Low insurance was identified among unspecified visa status (aOR= 0.02). |  |
| Nagai et al., 2023^5^ | 182 Brazilian adult immigrants residing in Shiga prefecture. | To assess Brazilian’s knowledge level of HIV/AIDS and the awareness of public health centers in Japan, and to explore factors related to these items. | Survey via internet and mail. | Perception of needs and desire for care | Facilitator: Most respondents were familiar with HIV/AIDS.  Barrier: Only 58% knew the existence of PHCs, and only 25% knew that HIV testing is available at PHCs free of charge and anonymously. Intermediate (OR: 5.70) and beginner Japanese proficiency (OR: 6.81) were less likely to be aware of public health centers than those with advanced proficiency. |  |
| Kobori et al., 2022^6^ | 516 Thai immigrants aged 20 years or older residing across Japan. | To clarify the status of regular dental checkups and related factors among Thai immigrants in Japan. | Online survey. | Healthcare utilization | Facilitator: The percentage of regular dental checkup was significantly higher among females, those in older age groups, those who had lived in Japan for a longer period, those with better economic conditions and those who were confident in their Japanese skills. |  |
| Okamoto et al., 2022^7^ | 209 immigrants excluding student visa holders residing in Greater Tokyo area. | To examine the healthcare needs and difficulties experienced by immigrants in Japan by level of their Japanese language fluency. | Face-to-face survey at medical facilities. | Healthcare consequences | Barrier: Those with less Japanese language fluency experienced statistically significant uneasiness in communication during clinic visits. |  |
| Yoshino et al., 2021^8^ | 342 adult Filipino women residing in Aichi prefecture | To investigate associations between social support and access to healthcare of Filipino women. | Self-administered questionnaire distributed in churches and a festival. | Healthcare reaching | Facilitator: Women with the highest emotional/informational (aOR=0.22) and tangible support score (aOR= 0.38) showed the low unmet need. Barrier: Women with a Japanese husband also showed lower odds of having a regular healthcare provider. (aOR=0.44) |  |
| Higuchi et al., 2021^9^ | 608 immigrants consulted with a non-governmental organization (NGO) residing in Aichi prefecture | To identify factors associated with “being advised to visit a medical facility” and “being referred to a medical facility,” which represented hypothetical proxy indicators of barriers to healthcare access. | Review of activity records of an NGO* that provides free consultations targeting immigrants. | Healthcare utilization | Barrier: Those who were not covered by public insurance (OR=1.56), unemployed people (OR=3.28), and students (OR=2.77) were more likely to be advised or referred to a medical facility. |  |
| Bhandari et al., 2020^10^ | 200 Nepalese immigrants residing in Tokyo prefecture | To evaluate the prevalence of cancer information seeking and scanning and to clarify the associated factors. | Self-administered questionnaire distributed through snowball sampling. | Perception of needs and desire for care, healthcare consequences | Facilitator: High education level and Japanese language skills were positively associated with both cancer information seeking and scanning. Migrants with low perceived health status were more likely to perform information scanning, while those who had been ill last year and who perceived proper access to doctors were more likely to undertake information seeking. |  |
| Shakya et al., 2020^11^ | 769 Chinese, Vietnamese, and Nepalese students from Japanese language schools in Shinjuku, Tokyo. | To examine the factors associated with access to HIV testing among international students in language schools. | Self-administered questionnaire distributed in schools. | Perception of needs and desire for care | Barrier: Nepalese students were less likely to know where to receive HIV tests compared to Chinese students (aOR =0.12).  Facilitator: Students with better language ability had more knowledge. (aOR=1.93) |  |
| Nakazawa et al., 2019^12^ | 59 immigrants from 27 developing countries with work or study experience in Japan (not specified region) | To identify the experiences of immigrants for the use of emergency medical services. | Questionnaire survey. | Perception of needs and desire for care, seeking and utilization | Facilitator: Main source of information for emergency healthcare through internet, friends and public announcements.  Barrier: They felt anxious about the language, healthcare cost, the treatment and culture. |  |
| Ohashi., 2019^13^ | 61 international students from 34 countries (not specified region) | To identify the current situation and issues on mental health of international students | Survey via mail. | Healthcare reaching and healthcare consequences | Barrier: Interpreter is required. Immigrants have limited social resources to prevent diseases. |  |
| Shah et al., 2018^14^ | 189 married female Nepalese immigrants residing in Tokyo, Kanagawa, Chiba, Saitama, Tochigi prefectures. | To explore the association between the use of modern contraceptive methods by Nepalese women and quality of life. | Survey via telephone. | Perception of needs and desire for care | Barrier: Low contraceptive knowledge was associated with limited access to modern contraceptives. (aOR=1.31) |  |
| Kinoshita et al., 2018^15^ | 78 HIV infected immigrant patients (with 473 Japanese patients) visiting the outpatient clinic of AIDS Clinical Center, National Center for Global Health and Medicine in Tokyo from 2011 to 2014. | To identify factors associated with retention in HIV/AIDS care among immigrant patients. | The review of clinic records. | Healthcare consequences | Barrier: Twelve out of thirteen lost-to-follow-up non-Japanese patients held legal status to reside in Japan and were eligible for public health services. Nine had limited fluency in Japanese language, and six used alternative verbal communication. |  |
| Shakya et al., 2018^16^ | 642 Nepalese immigrants residing in across Japan. | To examine factors associated with access to healthcare among Nepalese immigrants. | Self-administered questionnaire distributed through snowball sampling. | Healthcare utilization and consequences | Barrier: Those who did not need interpreter during medical visits (aOR=0.34) and irregular payment of health insurance were associated with not seeing a doctor. (aOR=4.09) |  |
| Yamamoto et al., 2017^17^ | 84 immigrants, from 23 countries residing mainly in Kanto and Chubu region, who consulted through telephone at an NGO from April 2023 to December 2015. | To determine the factors that inhibit immigrants from receiving HIV testing and treatment. | The review of activity records of immigrants who consulted at an NGO*. | Perception of needs and desire for care, utilization and consequences | Barrier: Highest number of inquiries were related to language barriers and request for translators (35.6%), followed by inquiries regarding treatment and care (25%), and then economic and social aspects of immigrants’ life (11.4%). |  |
| Hashimoto et al., 2017^18^ | 558 Brazilian mothers of school-going children residing in Aichi, Mie and Shizuoka prefectures. | To develop a health literacy scale among Brazilian mothers in Japan. | Self-administered questionnaire distributed via schools. | Perception of needs and desire for care | Facilitator: Longer stay, higher education level, and Japanese language proficiency, were associated with health literacy. |  |
| Tsuda et al., 2015^19^ | 159 immigrants who were diagnosed with tuberculosis residing in Osaka city. | To analyze pulmonary tuberculosis treatment outcomes among foreign nationals of different backgrounds. | The review of patient registry. | Healthcare utilization and consequences | Barrier: The default rate among smear-negative cases of 14.5%. The rate of overseas transfer out (44.4%) was higher among patients not covered by health insurance. |  |
| Takaku et al., 2015^20^ | 245 Spanish speaking Latin American immigrants mainly with permanent and long-term visas residing in Aichi prefecture | To explore the factors associated with information-seeking behaviors regarding medical institutions with Spanish language support. | Self-administered questionnaire distributed through public events. | Perception of needs and desire for care, healthcare seeking | Information-seeking behavior was associated with having previously experienced a need for Spanish language support when seeing doctors in Japan, finding and attending medical institutions with Spanish language support in the Tokai area, length of residency in Japan, poor Japanese language skills, and the Spanish language used in daily life. |  |
| Kadota et al., 2014^21^ | 826 reported immigrant tuberculosis cases in public health centers across Japan. | To elucidate the problems that compel immigrants to return to their country partway through treatment. | Review of data from the public health centers. | Healthcare reaching, utilization and consequences | Barrier: Immigrants with duration of stay less than three years (24.3%) and illegal residents (26.5%), those without health insurance (33.6%) returned to the country.  Facilitator: A smaller percentage of migrants living in metropolitan area returned to the country during treatment. |  |
| Nakao et al., 2013^22^ | 61 immigrants with HIV infection residing across Japan who contacted two non-governmental organizations. | To clarify the factors preventing HIV-positive immigrants from receiving early medical examinations. | Review of consultation records of an NGO*. | Perception of needs and desire for care, healthcare utilization, consequences | Barrier: Language issues (33.3%), and issues with access to health insurance (17.2%) were the main consultation topics. |  |
| Igarashi et al., 2013^23^ | 236 immigrant mothers (mainly from China, South Korea, Philippines and Brazil) who experienced delivery within the past 2-4 days in 16 hospitals across Japan (With 568 Japanese mothers) | To evaluate maternity care from the perspectives of immigrant women. | Face-to-face survey at hospitals. | Healthcare consequences | Barrier: Some Japanese literacy obstructed positive communication and enhanced loneliness during delivery. (Mean=0.34) |  |
| Fujita et al., 2013^24^ | 50 immigrant men from mainly European countries, Australia and USA residing in Kyoto prefecture. | To determine factors that influenced foreign residents' dental treatment choices in Japan | Face-to-face survey at clinics. | Healthcare seeking | Facilitator: The clinics they visited were recommended by foreign acquaintances, and the common features of these clinics were that the doctors spoke English and were well prepared to accept foreign patients. |  |
| Suguimoto et al., 2012^25^ | 282 randomly selected documented Latin American immigrants from Nagahama city (Kansai region) | To assess the pattern of health insurance coverage and predictors of uninsurance. | Face-to-face survey. | Healthcare utilization | Barrier: No chronic disease (aOR=19.8), no knowledge of health insurance (aOR=6.36) and poor education (aOR=3.72) were associated with uninsurance. |  |
| Martinez et al., 2008^26^ | 133 Latin American immigrants residing in Shiga prefecture | To examine the status of medical insurance enrollment and medical response behavior. | Self-administered questionnaire. | Healthcare utilization and consequences | Barrier: The most common reason for not having insurance was "high insurance premiums." When feeling unwell, 35% of respondents said they "went to the hospital," 17% said they "took time off work/school," and 15% said they "took medicine brought from their home country." |  |
| Reshad et al., 2008^27^ | 220 immigrants coming to a clinic in Shimada, Shizuoka prefecture from 1993 to 2007 | To identify health and social situations of immigrants in Japan. | Review of clinic data. | Healthcare utilization | Barrier: The rate of continued consultation for chronic disease was higher among those with insurance (60.6%) compared to those without insurance (21.6%). |  |
| Takahashi et al., 2007^28^ | 9 Brazilian women who gave birth in Shiga prefecture | To investigate the current state of maternal and childcare during pregnancy, delivery, and the postpartum period. | Self-administered questionnaire | Healthcare reaching and consequences | Barrier: Not having an interpreter. Facilitator: They used foreign language textbooks and mother and child health handbooks in their native language. The main supporters during delivery were their husbands and families. They were satisfied with the medical care during delivery. |  |
| Isono et al., 2004^29^ | 146 parents of foreign children (mainly Chinese and Korean) residing in A Ward, Tokyo. | To examine vaccination status of foreign children and their caregivers’ awareness of vaccinations and childcare. | Self-administered questionnaire distributed at nurseries. | Perception of needs and desire for care | Facilitator: Information about vaccinations was mainly obtained through postcards from the public health center, and 68% of parents had received an explanation about getting their children vaccinated. Over 90% of parents answered that the reason they wanted their children vaccinated was “because it is necessary for their child’s health.” |  |
| Kojima., 2004 ^30^ | 497 Brazilian immigrant workers residing in Iwata City (Chubu region) | To examine determinants of health insurance coverage, medical care behaviors and troubles with medical care facilities. | Self-administered questionnaire | Healthcare utilization | Facilitator: Ability to collect information and communicate (fluency in Japanese), the necessity of health and medical services and the type of employment affected health insurance coverage and care behaviors. |  |
| Gu et al., 2004^31^ | 98 Chinese women students attending at the University of Tokyo | To explore the medical and health needs. | Self-administered questionnaire survey. | Perception of needs and desire for care and healthcare consequences | Barrier: Chinese women faced language barriers and required translators and medical guidebooks in Chinese to obtain healthcare. They also lacked knowledge about the Japanese medical and health system. |  |
| Ito et al., 2004^32^ | 82 Latin American mothers residing in Komaki city, Aichi prefecture and review of hospital records from 1993 to 2000 | To identify the communication barrier between healthcare provider and recipient | Self-administered questionnaire distributed at hospital and review of hospital records. | Healthcare consequences | Facilitator: 95.1% were satisfied with healthcare. 24.1% were able to communicate with healthcare providers. The deployment of interpreters led to a 6.3-fold increase in the number of users of maternal and child health care services. |  |
| Sasagawa et al., 2004^33^ | 43 Latin American women who have ever become pregnant and given a birth in Japan (not specified region) | To identify maternal and child health service utilization among Latin American women. | Self-administered questionnaire. | Healthcare consequences | Facilitator: The attitude of doctors and nurses was associated with their wish for delivery at the same hospital. |  |
| Yamamura et al., 2002^34^ | 23 undocumented immigrants with HIV infection who consulted at a clinic in Yokohama | To identify the factors and issues to deal with HIV infected immigrants in Japan. | Review of clinic records. | Healthcare reaching and utilization | Barrier: Due to their legal status, their access to medical information and insurance was restricted. |  |
| Inoue et al., 2000^35^ | 87 immigrant pregnant women who gave birth at a health center in Tokyo | To examine the status of antenatal checkups and the influencing factors. | Review of clinic records. | Healthcare reaching, utilization and consequences | Barrier: Mothers in the group receiving less than 75% of antenatal care had poor Japanese language (N=9, 60.0%), no health insurance (N=12, 80%), and the foreign husband (N=10, 90.9%). |  |
| Anami et al.,1999^36^ | 15 Brazilian and Peruvian mothers from a health center (not specified region) | To examine the situations of immigrant mothers and children during perinatal period | Self-administered questionnaire distributed at hospital | Healthcare consequences | Barrier: 64.3% had experienced problems due to differences in language and customs, and communication problems with medical professionals were particularly common. |  |
| Momose et al.,1995^37^ | 37 international university students (With 253 medical doctors) residing in Fukuoka prefecture | To analyze the problems in medical care for immigrants. | Self-administered questionnaire distributed via mail. | Perception of needs and desire for care, utilization and consequences | Barrier: Only 12.4% of patients were accompanied by interpreters. Foreign students found it difficult to obtain information on the city’s medical system. 6.3% of medical doctors had an experience of unpaid medical bills. |  |
| Watanabe et al., 1995^38^ | 82 English-speaking immigrant women who had experienced pregnancy and childbirth in Japan residing around Tokyo prefecture. | To clarify the actual utilization of maternal and child health care and medical care in Japan, and to identify problems and needs. | Self-administered questionnaire distributed via mail. | Healthcare consequences | Barrier: High satisfaction (93%) regarding giving birth in Japan but complaints were about long waiting times, insufficient facility and explanation. |  |
| Kunii et al., 1993^39^ | 371 immigrant factory workers from Tochigi prefecture. | To reveal certain health issues and illness behaviors among immigrants. | Self-administered questionnaire distributed via mail. | Healthcare reaching and utilization | Barrier: Heavy work duties, language barriers, and high medical costs were the top reasons for not going to clinics when necessary. |  |
| Saito et al., 1992^40^ | 69 immigrant mothers (mainly Chinese, Koreans and Philippines) residing in Tokyo who experienced delivery in Japan (with 325 Japanese mothers) | To understand the level of maternal and child health care and problems of immigrants in Japan. | Self-administered questionnaire distributed at a hospital. | Perception of needs and desire for care | Barrier: Knowledge about healthcare access was lower among immigrant mothers who were born in foreign countries, had foreign husbands, had poor Japanese language, and had childbirth in a foreign country. |  |

OR= Odds ratio, aOR=adjusted odd ratio, RR= relative risk, N=numbers

| Supplementary Table 2: Characteristics of qualitative studies (N=23) | | | | | | |
| --- | --- | --- | --- | --- | --- | --- |
| First author and year of publication | Study population (Sample size, country of origin, type of visa (if applicable) and the location of study) | Objectives | Method of data collection | Stage of access to healthcare | Main results |  |
| Mori et al., 2022^41^ | 13 international faculty staff, and students from Okinawa Institute of Science and Technology Graduate University | To understand the difﬁculties faced by highly skilled immigrants when dealing with the Japanese healthcare system. | Semi-structured interviews. | Healthcare consequences | Barrier: Japanese language communication, flow, and procedures in the hospital and building open relationships with doctors were challenging. |  |
| Matsuoka et al., 2022^42^ | 34 Vietnamese, Myanmar, and Nepalese international students studying at Japanese-language schools or vocational/technical schools, technical intern trainees, and cooks for  restaurants and their spouses residing in Tokyo | To explore the barriers and facilitators for immigrant access to health-related information, health services, and welfare services during the ﬁrst wave of COVID-19. | Semi-structured interviews and focus group discussions. | Perception of needs and desire for care | Barrier: Language was the barrier.  Facilitator: Social media, short information provided using native language or simple Japanese using illustrations and supportive people were facilitators for access to health-related information and services. |  |
| Horimoto et al., 2022^43^ | 12 Vietnamese technical intern trainees residing in Kansai region | To identify difficulties encountered in receiving medical care. | Semi-structured interviews. | Healthcare reaching | Barrier: Inability to seek information, and hesitation to seek medical help due to work and scheduling were also present.  Facilitator: Support from company or supervising organization officials was required. |  |
| Kohno et al., 2022^44^ | 45 Muslims from Indonesia and Malaysia residing in Kansai region | To identify factors influencing healthcare-seeking behaviors of Muslim immigrants and to explore issues with healthcare experiences. | Semi-structured interviews. | Perception of needs and desire for care, healthcare seeking | Barrier: Religion, confusion about the healthcare system compared to home country, and language barrier problems determined health-seeking.  Facilitator: Muslim friends living in the same community are supportive to healthcare seeking. |  |
| Nagamine et al., 2022^45^ | 18 immigrants (Long term resident visa, technical intern visa, student visa) who have concerns about Japanese language ability and have visited Japanese medical institutions (not specified region) | To identify and structure difficulties caused by communication based on the experiences of immigrant patients | Semi-structed interviews. | Healthcare seeking and consequences | Barrier: Lack of patient-centered communication and intercultural competence among health care providers, one-sided use of machine translation by health care providers, lack of information about Japanese medical institutions and culture, over expectations for Japanese health care, structural problems of foreign community and lack of interpreters that met patient expectations caused communication problems. |  |
| Khin et al., 2021^46^ | 17 married Myanmar women (with 4 interpreters) residing in greater Tokyo area. | To explore perceived barriers to access, with a speciﬁc focus on contraceptive services, and their consequences. | Semi-structured interviews. | Perception of needs and desire for care, healthcare seeking, reaching, utilization and consequences | Barrier: Language barriers, limited information sources, health beliefs, and cultural and ﬁnancial factors affected access at different stages. |  |
| Suzuki et al., 2021^47^ | 4 Peruvian mothers (not specified region) | To clarify difficulties Peruvian mothers, face when seeing a doctor for their children. | Semi-structured interviews. | Healthcare seeking, reaching, and consequences | Barriers: They had difficulties taking their children to medical institutions due to differences in language, medical systems and cultural backgrounds, lack of support, and not being able to take time off work. They expressed dissatisfaction when medical professionals did not understand and allowed to speak their thoughts. |  |
| Soneta et al., 2021^48^ | 9 international students from various countries studying at a medical university in Tokyo | To describe the experience of international students who visited hospitals in Japan | Semi-structured interviews. | Healthcare consequences | Facilitators: Participants were satisfied with an efficient medical system and kind staff. Barriers: They had difficulties due to language and cultural differences. |  |
| Tachibana et al., 2020^49^ | 6 mid-to-long term residents with type 2 diabetes from a hospital and a clinic (not specified region) | To clarify the experiences of mid-to-long-term immigrants of Japan with type 2 diabetes. | Semi-structured interviews. | Healthcare consequences | Barrier: Differences in culture and health management affected the management of Type 2 Diabetes. |  |
| Sudo et al., 2019^50^ | 24 foreign mothers residing in five prefectures with largest immigrant population | To elucidate the health behaviors of foreign mothers in Japan regarding their children and the factors that affected these behaviors. | Focus group discussions. | Perception of needs and desire for care, healthcare seeking | Facilitator: Mainly gathered information about child health from family, friends, internet and SNS. Selected adequate healthcare facilities through convenience, quality of healthcare provider and English ability of the medical doctor mainly. |  |
| Ota et al., 2019^51^ | 36 women immigrants, aged 20 and older who visited an Islamic religious institution (mosque) from prefecture A (not specified region) | To clarify the needs of health information by Muslim women. | Participant observations and key-informant interviews | Perception of needs and desire for care | Facilitator: Community with immigrant and native Muslim women supported the health information. |  |
| Nakano et al., 2019^52^ | 5 Chinese mothers from hospital A (not specified region). | To examine the postpartum care needs of Chinese mothers in Japan. | Semi-structured interviews. | Healthcare consequences | Barrier: Lack of cultural sensitivity of health care providers.  Facilitator: High satisfaction with childbirth in Japan. |  |
| Ohira et al., 2018^53^ | 9 Chinese women immigrants (not specified region). | To clarify the perception of Chinese mothers in Japan regarding maternal and child health and childcare support services. | Semi-structured interviews. | Healthcare consequences | Barrier: Difficulty in communication due to language and culture.  Facilitator: Chinese mothers were satisfied with healthcare. |  |
| Teraoka et al., 2017^54^ | 22 immigrants (not specified region) | To clarify aspects of cross-cultural experience perceived by immigrants. | Focus group discussions. | Healthcare seeking and consequences | Barrier: Complicated medical systems, language, insufficient communication, different cultures, and not relying on nurses were the barriers to use healthcare services. |  |
| Watanabe., 2017^55^ | 13 immigrants from mainly English-speaking countries residing in Fukui prefecture | To investigate communication difficulties between immigrants and the healthcare institutions. | Narrative interviews. | Healthcare seeking, healthcare consequences | Barrier: Privacy and confidentiality, cultural differences, differences in the medical system, and language-related issues were concerns for receiving healthcare among participants. |  |
| Dodo et al., 2013^56^ | 4 Chinese women with spouse visa in Yamagata prefecture | To explore the problems and solutions on the acceptance of immigrants at medical institutions. | Focus group discussions. | Healthcare seeking and consequences | Barrier: Differences in healthcare systems, communication, and dissatisfaction towards healthcare providers and treatment developed during the experiences of receiving healthcare. |  |
| Umemura et al., 2012^57^ | 10 Brazilian expectant mothers with poor Japanese, and those who were delivering for the 1^st^ time in Japan (not specified region) | To examine the health care needs of Brazilian pregnant and nursing mothers in Japan. | Participant observations and informal interviews. | Healthcare seeking | Barrier: Women were confused about systems and customs that were different from their country of origin. |  |
| Hashimoto et al., 2011^58^ | 18 immigrant women who have experience of delivery or child raising in Japan (not specified region) | To identify the difficulties during perinatal care in Japan and strategies to overcome these difficulties. | Semi-structured interviews. | Healthcare reaching | Facilitator: Women’s efforts, social support, and the use of interpreters lead to the use of the Japanese healthcare system. |  |
| Nagata et al., 2010^59^ | 18 Brazilians residing in the central and western areas of Shizuoka prefecture and who have used medical services themselves or for their family member. | To clarify the issues surrounding the use of makeshift interpreters | Focus group discussions | Healthcare consequences | Barrier: Problems with using makeshift interpreters included inaccuracy, lack of knowledge of terminology, time taken to find makeshift interpreters, resulting in patients not understanding the doctor's explanation, not being able to give enough information to the doctor. |  |
| Sugiura., 2009^60^ | 29 Brazilian women in Japan and their families, 7 medical interpreters, and 13 nurses from an obstetric clinic (not specified region). | To clarify the perceptions of Brazilian women in Japan who are raising children toward maternal and child health care in Japan | Semi-structured interview and participant observations at an obstetric clinic | Perception of needs and desire for care and consequences | Barrier: Due to the language barrier and limited information, the Brazilian women were very worried about how to respond if their children suddenly fell ill. These women had dissatisfaction about Japan healthcare due to health system differences and drug prescription which was not effective for them. |  |
| Itoi, 2008^61^ | 26 Cambodians who can speak Japanese (With 4 Japanese who support them) residing in Kanto region | To understand the　characteristics　of　Cambodian immigrants’ feelings　when　attending　Japanese　healthcare　centers. | Interviews and Leininger’s　observation-participation-reflection　process. | Healthcare consequences | Barrier: Language, different dietary and cultural habits, shame for genital examination, and the burden of medical expenses were felt while attending healthcare centers. |  |
| Kubota et al., 2004^62^ | 9 Brazilian couples who gave birth at one hospital and two clinics (not specified region). | To clarify the current situation and problems during perinatal period. | Semi-structured interview repeated twice during peri-natal period. | Perception of needs and desire for care, healthcare reaching | Facilitator: All nine couples were unable to understand perinatal technical terms, so they visited the clinic accompanied by a friend or an interpreter. Information during pregnancy was obtained from mothers' classes, friends, Portuguese magazines, the Internet. |  |
| Kitada et al, 1993^63^ | 18 immigrant women mainly from the Philippines (not specified region). | To clarify the problems of childbirth and child-rearing from the perspective of foreigners in Japan, | Interview. | Perception of needs and desire for care, and healthcare consequences | Barrier: Six of the 14 women who gave birth in Japan attended the mother's class, and they evaluated it as useful for first-time births, and the others attending because they did not understand Japanese.  Facilitator: They learned about the existence of the health center from postcards informing them about health checkups, or from their husbands, mothers-in-law, or friends. |  |

| Supplementary Table 3: Characteristics of mixed-methods studies (N= 4) | | | | | | |
| --- | --- | --- | --- | --- | --- | --- |
| First author and year of publication | Study population (Sample size, Country of origin and type of visa) | Objectives | Location and method of data collection | Stage of access to healthcare | Main results |  |
| Lee et al., 2023^64^ | 165 Vietnamese adult immigrants and 3 key-informants from Tokyo prefecture. | To explore the health issues and health-related behaviors of Vietnamese immigrants. | Questionnaire survey followed by key-informant interviews. | Perception of needs and desire for care | Barrier: People who contacted family members living in Vietnam or overseas using social networking services (SNS) when needed to consult someone about their health (aOR=6.09) were more likely to present with one or more of the typical TB symptoms.  Facilitator: Key informant interviews revealed language barriers and recent migration hinder health-seeking behaviors. |  |
| Paudel et al., 2023^65^ | 89 Nepalese adult immigrants across Japan for focus group discussions and 937 respondents for online survey. | To identify barriers to healthcare access due to the COVID-19 crisis. | Focus group discussions followed by online survey. | Perception of needs and desire for care, healthcare seeking, reaching and utilization, consequences | Twenty-six themes on barriers and six on facilitators were identified by the focus group discussions, among which 17 have significant associations in logistic regression.  Barrier: Financial barriers, lack of information and awareness of available services, perceived delay in care (OR = 0.63), limited operating hours of the hospital (OR = 0.60), perceived complexity in vaccine registration (OR = 0.60), lack of medical interpreters (OR = 0.70), lack of hotline services (OR = 0.67), language barriers (OR = 0.51), communication barriers (OR = 0.55), inefficiency of low dose drug (OR = 0.67), unfamiliarity with Japanese medical system (OR = 0.52), fear of losing job (OR = 0.74), fear of discrimination (OR = 0.57) and legal barriers (OR = 0.61) were the barriers to access healthcare during COVID-19.  Facilitator: Information from Nepali doctors/ nurses and healthcare volunteers was the facilitator (OR = 1.36) |  |
| Morita et al., 2021^66^ | 522 immigrants (mainly from China/Taiwan, Nepal, Philippines, Vietnam (including refugees and asylum seekers)) residing in Kanto region followed by interviews with 11 immigrants and 3 Japanese supporters | To obtain information on the characteristics of immigrants who had difficulties in accessing healthcare, factors that inhibit access to care, and effective ways to help them. | Secondary data of structured interview survey followed by semi-structured interviews. | Perception of needs and desire for care, healthcare seeking, reaching and utilization | Barriers: The proportion of those who refrained from seeking medical care was higher among men, single people, people living alone, and people from low-income households, and by type of residence status, it was more prevalent among international students, technical intern trainees, and people with specific activities (refugee recognition applicants). Not having health insurance or being an irregular resident was strongly associated with refraining from seeking medical care.  Facilitators: Qualitative research revealed that communities and networks of supporters are helpful in situations where access to medical care is difficult, such as not having health insurance or being an irregular resident. |  |
| Nagamatsu et al., 2020^67^ | 35 immigrants mainly from the Philippines residing in Chiba and Yamagata prefecture | To clarify the risks and perceptions of non-communicable diseases and health promotion behavior of middle-aged female immigrants in Japan. | Self-administered questionnaire followed by focus group discussions. | Healthcare consequences | 80% received regular health check-up, 49% received breast cancer screening, and 34% received cervical cancer screening.  Barriers: The monolingual Japanese health service prevented immigrant women from understanding their health check-up and cancer screening results, and how to utilize the health service system.  Facilitator: They recognized the threat of non-communicable diseases |  |

OR= Odds ratio, aOR=adjusted odd ratio

Supplementary Table 4: Quality Assessment of quantitative studies based on Mixed Method Appraisal Tool (MMAT)^68^

| Author | 4.1 | 4.2 | 4.3 | 4.4 | 5.4 |
| --- | --- | --- | --- | --- | --- |
| Teng et al., 2023^1^ | Y | Y | Y | N | Y |
| Teng et al., 2023^2^ | Y | Y | Y | N | Y |
| Iglesias Rodriguez et al., 2023^3^ | Y | Y | Y | C | Y |
| Supakul et al., 2023^4^ | Y | N | Y | Y | Y |
| Nagai et al., 2023^5^ | Y | N | Y | Y | Y |
| Kobori et al., 2022^6^ | Y | Y | Y | Y | Y |
| Okamoto et al., 2022^7^ | Y | Y | Y | C | Y |
| Yoshino et al., 2021^8^ | Y | N | Y | C | Y |
| Higuchi et al., 2021^9^ | Y | Y | Y | Y | Y |
| Bhandari et al., 2020^10^ | Y | Y | Y | C | Y |
| Shakya et al., 2020^11^ | Y | Y | Y | N | Y |
| Nakazawa et al., 2019^12^ | Y | N | N | N | Y |
| Ohashi., 2019^13^ | C | N | Y | N | N |
| Shah et al., 2018^14^ | Y | Y | Y | Y | Y |
| Kinoshita et al., 2018^15^ | Y | N | Y | Y | Y |
| Shakya et al., 2018^16^ | Y | Y | Y | Y | Y |
| Yamamoto et al., 2017^17^ | Y | N | N | Y | Y |
| Hashimoto et al., 2017^18^ | Y | Y | Y | Y | Y |
| Tsuda et al., 2015^19^ | Y | Y | Y | Y | Y |
| Takaku et al., 2015^20^ | Y | Y | N | Y | Y |
| Kadota et al., 2014^21^ | Y | Y | Y | Y | Y |
| Nakao et al., 2013^22^ | Y | N | N | Y | Y |
| Igarashi et al., 2013^23^ | Y | Y | Y | N | Y |
| Fujita et al, 2013^24^ |  |  |  |  |  |
| Suguimoto et al., 2012^25^ | Y | Y | Y | Y | Y |
| Martinez et al., 2008^26^ | Y | Y | N | N | Y |
| Reshad et al., 2008^27^ | Y | Y | N | N | Y |
| Takahashi et al., 2007^28^ | Y | N | N | N | Y |
| Isono et al., 2004^29^ | Y | Y | N | N | Y |
| Kojima., 2004 ^30^ | Y | N | C | N | C |
| Gu et al., 2004^31^ | Y | N | Y | N | Y |
| Ito et al., 2004^32^ | Y | N | N | Y | Y |
| Sasagawa et al., 2004^33^ (Conference) | N | N | N | N | N |
| Yamamura et al., 2002^34^ | Y | N | Y | Y | Y |
| Inoue et al., 2000^35^ | Y | N | Y | Y | Y |
| Anami et al.,1999^36^ (Conference) | Y | N | N | N | Y |
| Momose et al.,1995^37^ | N | N | Y | N | Y |
| Watanabe et al., 1995^38^ | Y | N | N | N | Y |
| Kunii et al., 1993^39^ | Y | Y | N | N | Y |
| Saito et al., 1992^40^ | Y | N | Y | Y | Y |

Supplementary Table 5: Quality Assessment of qualitative studies based on Mixed Method Appraisal Tool (MMAT) ^68^

| Author | 1.1 | 1.2 | 1.3 | 1.4 | 1.5 |
| --- | --- | --- | --- | --- | --- |
| Mori et al., 2022^41^ | Y | Y | Y | Y | Y |
| Matsuoka et al., 2022^42^ | Y | Y | Y | Y | Y |
| Horimoto et al., 2022^43^ | Y | Y | Y | Y | Y |
| Kohno et al., 2022^44^ | Y | Y | Y | Y | Y |
| Nagamine et al., 2022^45^ | Y | Y | Y | N | Y |
| Khin et al., 2021^46^ | Y | Y | Y | Y | Y |
| Suzuki et al., 2021^47^ (Conference) | Y | Y | Y | Y | Y |
| Soneta et al., 2021^48^ | Y | Y | Y | Y | Y |
| Tachibana et al., 2020^49^ | Y | Y | Y | Y | Y |
| Sudo et al., 2019^50^ | Y | Y | Y | Y | Y |
| Ota et al., 2019^51^ | Y | Y | Y | Y | Y |
| Nakano et al., 2019^52^(Conference) | Y | Y | C | N | Y |
| Ohira et al., 2018^53^(Conference) | Y | Y | C | N | Y |
| Teraoka et al., 2017^54^ | Y | Y | Y | Y | Y |
| Watanabe., 2017^55^ | Y | Y | Y | N | Y |
| Dodo et al., 2013^56^ | Y | Y | Y | N | Y |
| Umemura et al., 2012^57^ | Y | Y | Y | Y | Y |
| Hashimoto et al., 2011^58^ | Y | Y | Y | Y | Y |
| Nagata et al., 2010^59^ | Y | Y | Y | N | Y |
| Sugiura., 2009^60^ | Y | Y | Y | Y | Y |
| Itoi, 2008^61^ | Y | Y | Y | N | Y |
| Kubota et al., 2004^62^ (conference) | Y | Y | Y | N | Y |
| Kitada et al, 1993^63^(conference) | Y | Y | N | N | Y |

Supplementary Table 6: Quality assessment of mixed-methods studies based on Mixed Method Appraisal Tool (MMAT) ^68^

|  | 5.1 | 5.2 | 5.3 | 5.4 | 5.5 |
| --- | --- | --- | --- | --- | --- |
| Lee et al., 2023 | Y | C | Y | N | Y |
| Paudel et al., 2023 | Y | Y | Y | Y | Y |
| Morita et al., 2021 | Y | Y | Y | N | N |
| Nagamatsu et al, 2020 | Y | Y | Y | N | N |

References

1. Teng Y, Hanibuchi T, Nakaya T. Does the Integration of Migrants in the Host Society Raise COVID-19 Vaccine Acceptance? Evidence From a Nationwide Survey in Japan. Journal of Immigrant and Minority Health. 2023;25(2):255-65.

2. Teng Y, Hanibuchi T, Machida M, Nakaya T. Psychological determinants of COVID-19 vaccine acceptance: A comparison between immigrants and the host population in Japan. Vaccine. 2023;41(8):1426-30.

3. Iglesias Rodríguez IM, Miura S, Maeda T, Imai K, Smith C, Vasquez Velasquez C, et al. Analysis of the Chagas disease situation in Japan: a cross sectional study and cost-effectiveness analysis of a Chagas disease screening program. The Lancet Regional Health - Western Pacific. 2023;31:100574-.

4. Supakul S, Jaroongjittanusonti P, Jiaranaisilawong P, Phisalaphong R, Tanimoto T, Ozaki A. Access to Healthcare Services among Thai Immigrants in Japan: A Study of the Areas Surrounding Tokyo. International Journal of Environmental Research and Public Health. 2023;20(13).

5. Nagai S, Kitahara T, Kito K, Hitosugi M. HIV/AIDS knowledge level, awareness of public health centers and related factors: a cross-sectional study among Brazilians in Japan. BMC Public Health. 2023;23(1):2379-.

6. Kobori E, Fukuda H, Hazano S, Maeda Y, Mizumoto K, Nozue M, et al. Web-based health research of Thai residents in Japan: examination of the representativeness of the surveyed population by data collection method. 国際保健医療. 2022;37(3):150-1.

7. Okamoto M, Matsuda Y, Foronda CL. Healthcare needs and experiences of foreign residents in Japan by language fluency. Public Health Nursing. 2022;39(1):103-15.

8. Yoshino A, Salonga RB, Higuchi M. Associations between social support and access to healthcare among Filipino women living in Japan. Nagoya Journal of Medical Science. 2021;83(3):551-.

9. Higuchi M, Endo M, Yoshino A. Factors associated with access to health care among foreign residents living in Aichi Prefecture, Japan: secondary data analysis. International Journal for Equity in Health. 2021;20(1):135-.

10. Bhandari D, Ozaki A, Kobashi Y, Higuchi A, Shakya P, Tanimoto T. Cancer information seeking and scanning behavior among Nepalese migrants in Japan and its association with preventive behavior. PLoS One. 2020;15(6):e0235275.

11. Shakya P, Sawada T, Zhang H, Kitajima T. Factors associated with access to HIV testing among international students in Japanese language schools in Tokyo. PLOS ONE. 2020;15(7):e0235659.

12. Nakazawa M, Sakaguchi M, Suzuki K, Ogawa M. Current status and issues of emergency medical services in Japan from the perspective of foreign residents in Japan: A consideration from survey results (In Japanese). Journal of Japanese Society for Emergency Medicine. 2019;22(2):340.

13. Ohashi T. Mental health of international students: current situation and issues (In Japanese). 2019.

14. Shah R, Kiriya J, Shibanuma A, Jimba M. Use of modern contraceptive methods and its association with QOL among Nepalese female migrants living in Japan. PLOS ONE. 2018;13(5):e0197243.

15. Kinoshita M, Oka S. Migrant patients living with HIV/AIDS in Japan: Review of factors associated with high dropout rate in a leading medical institution in Japan. PloS one. 2018;13(10):e0205184.

16. Shakya P, Tanaka M, Shibanuma A, Jimba M. Nepalese migrants in Japan: What is holding them back in getting access to healthcare? PLOS ONE. 2018;13(9):e0203645.

17. Yamamoto Y, Sawada T, Hirono F, Tarui M, Tsuyama N, Norimitsu S, et al. Factors that Inhibit Migrants Living in Japan from Receiving HIV Services: Analysis of Telephone Consultation Received by an NGO. Journal of International Health. 2017;32(3):158.

18. Hashimoto H, Yanagisawa S. Development of health literacy scale among Brazilian mothers in Japan. Health Promotion International. 2017;32(6):1034-40.

19. Tsuda Y, Matsumoto K, Komukai J, Kasai S, Warabino Y, Hirota S, et al. Pulmonary tuberculosis treatment outcome among foreign nationals residing in Osaka city. Kekkaku:[Tuberculosis]. 2015;90(3):387-93.

20. Takaku M, Ichikawa S, Kaneko N. A quantitative analysis of information-seeking behaviors regarding medical institutions with Spanish language support among South American Spanish-speaking migrants in Aichi Prefecture, Japan. Nihon Koshu Eisei Zasshi. 2015;62(11):684-93.

21. Kadota J, Kohno S, Amitani R, Keicho N, Takeyama H, Chonabayashi N, et al. Report from the committee of the Japanese Society for tuberculosis: A study of tuberculosis among foreigners resident in Japan, 2008: With Particular Focus on Those Leaving Japan in the Middle of Treatment. Japan Journal of Tuberculosis. 2014;89(1):5-12.

22. Nakao T, Yamamoto Y. Analysis of Factors Inhibiting Documented Migrants and Asylum Seekers from Accessing HIV Treatment in Japan, and Recommendations for Improving Their Access (In Japanese). Journal of the Japan Academy for Health Behavioral Science. 2013;28(1):105-14.

23. Igarashi Y, Horiuchi S, Porter SE. Immigrants’ Experiences of Maternity Care in Japan. Journal of Community Health. 2013;38(4):781-90.

24. Fujita J, Schoenbrunn E, Macmugen B. The Criteria for Choice of Dental Treatment: A Follow-up Survey of Foreign Residents. Journal of Oral Tissue Engineering. 2013;11(2):172-9.

25. Suguimoto SP, Ono-Kihara M, Feldman MD, Kihara M. Latin American immigrants have limited access to health insurance in Japan: a cross sectional study. BMC public health. 2012;12:1-9.

26. Martinez M, Matsuo T, Kawai Y, Hatashita H. Medical insurance and medical care behavior of South American foreign residents living in Shiga Prefecture: A survey of the actual medical needs of foreign residents in Shiga Prefecture (In Japanese). Journal of Nursing, Shiga University of Medical Science. 2008;6(1):54-8.

27. Reshad K, Maesato K. The Health and Social Situation of the Foreigners Living in Japan (In Japanese). Journal of International Health. 2008;23(1):15-7.

28. Takahashi K, Shigeta M, Nakamura Y, Lee S, Mashimo N, Nakata M, et al. A needs survey of medical services for foreign residents in Japan from the viewpoint of medical practitioners- Report of a survey for doctors in Gunma medical association and Gunma Pediatric association (In Japanese). Journal of International Health. 2010;25(3):181-91.

29. Isono F, Suzuki M, Ushijima K. Vaccination status of foreign children attending nursery schools and their caregivers' awareness of vaccinations and childcare (In Japanese). The Journal of Child Health. 2004;63(5):563-9.

30. Kojima H. Foreign Workers and Health Insurance in Japan: The Case of Japanese Brazilians. The Japanese Journal of Population. 2004;4.

31. Gu Y-H, Lee S, Ushijima H. A Study on the Needs of Medical, Maternal and Child Health Care in Chinese Women Students at the University of Tokyo. The Tohoku Journal of Experimental Medicine. 2004;204(1):71-8.

32. Ito M, Nakamura Y, Kobayashi A. The role of interpreters in maternal and child health for foreign residents in Japan (In Japanese). The Journal of Child Health. 2004;63(2):249-55.

33. Sasagawa E, Misago C. Study on maternal and child health service utilization among Latin American women living in Japan with focus on pregnancy and childbirth. 国際保健医療. 2004;18(1):131.

34. Yamamura J, Sawada T. The Actual Conditions and Medical Problems of HIV Patients Who Are Foreigners Having Overstayed Their Visas in Japan (In Japanese). The Journal of AIDS Research. 2002;4(2):53-61.

35. Inoue C, Lee S, Sato M. Examining the status of antenatal health checkups for foreign pregnant women residing in Japan (In Japanese). Journal of Japan Academy of Midwifery. 2000;13(3):162-7.

36. Anami M, Ueii T, Sato C. Maternal and Child Health of Foreign Residents in Japan (In Japanese). Japanese Journal of Maternal Health. 1999;40(3):288.

37. Momose Y, Esaki H. Characteristics of the Problems in Medical Care for Foreigners Living in Fukuoka City (In Japanese). Japanese Journal of Health and Human Ecology. 1995;61(6):336-47.

38. Watanabe Y, Higurashi M, Nakamura Y. What foreigners living in Japan want from maternal and child health and medical care in Japan (In Japanese). Journal of Maternal Health. 1995;36(2):337-42.

39. Kunii O, Nomiyama K. Present Status of Medical Care for Foreigners in Tochigi Prefecture, Japan (2) Illness Behavior of Foreign Workers (In Japanese). Japan Journal of Hygiene. 1993;48(3):685-91.

40. Saito T, Yoshioka T, Nakamura T, Noda A, Nakanishi Y, Kanemoto Y. Maternal and Child Health among Aliens Living in Tokyo (In Japanese). The Journal of Child Health. 1992;51(6):749-52.

41. Mori T, Deasy Y, Kanemoto E, Nakazawa E, Akabayashi A. Narratives on the Current Medical Situation in Japan According to Highly Specialized Foreign Professionals. Healthcare. 2022;10(9):1694-.

42. Matsuoka S, Kharel M, Koto-Shimada K, Hashimoto M, Kiyohara H, Iwamoto A, et al. Access to Health-Related Information, Health Services, and Welfare Services among South and Southeast Asian Immigrants in Japan: A Qualitative Study. International Journal of Environmental Research and Public Health. 2022;19(19):12234-.

43. Horimoto C, Uesugi Y. Difficulties Vietnamese Technical Intern Trainees Face in Obtaining Medical Services in Japan (In Japanese). Journal of International Health. 2022;37(1):1-9.

44. Kohno A, Dahlui M, Koh D, Dhamanti I, Rahman H, Nakayama T. Factors influencing healthcare-seeking behaviour among Muslims from Southeast Asian countries (Indonesia and Malaysia) living in Japan: an exploratory qualitative study. BMJ Open. 2022;12(10):e058718-e.

45. Nagamine M, Mori Y, Ohyama Y. Structuring Communication Difficulties of Foreign Residents When Visiting a Medical Institution in Japan. The Kitakanto Medical Journal. 2022;72(1):9-22.

46. Khin YP, Nawa N, Fujiwara T, Surkan PJ. Access to contraceptive services among Myanmar women living in Japan: A qualitative study. Contraception. 2021.

47. Suzuki M. Behavior and difficulties of children seeking medical care among Peruvian mothers in Japan (In Japanese). The Journal of Child Health. 2021;80(Lectures):189.

48. Soneta M, Kondo A, Abuliezi R, Kimura A. International students’ experience with health care in Japan. Sage Open. 2021;11(2):21582440211009211.

49. Tachibana R, Hatanaka K, Kawai N, Kitamura H, Shimizu Y. Experiences of Mid-to-long-term Residents with Type 2 Diabetes in Japan: A Qualitative Interview-based Study (In Japanese). Journal of Japan Academy of Nursing Science. 2020;40(0):661-71.

50. Sudo K, Hamamoto Y. Health behaviors of foreign mothers in Japan regarding their young children and the factors that affect these behaviors: A qualitative study. Japan journal of nursing science. 2019;16(4):420-32.

51. Ota K, Furusho H, Mawaki A, Niimi Y, Ikegami C, Arakawa N, et al. Health Information Sharing Among Muslim Women in a Japanese Mosque. MEDINFO 2019: Health and Wellbeing e-Networks for All: IOS Press; 2019. p. 1968-9.

52. Nakano M, Kuwano N, Maruyama K. A study on postnatal care needs of Chinese mothers who gave birth in Japan (In Japanese). Japanese Journal of Maternal Health. 2019;60(3):308.

53. Ohira M, Gao C. Chinese mothers' perceptions of Japan's maternal and child health and childcare support services (In Japanese). Journal of Maternal Health. 2018;59(3):322.

54. Teraoka M, Muranaka Y. Aspects of Cross-cultural Experience Perceived by Foreigners Living in Japan When Using Its Healthcare Services (In Japanese). Japan Journal of Academic Nursing Science. 2017;37:35-44.

55. Watanabe A. Listening to foreign patient voices: A narrative approach. Fukui Prefectural University Department of Medicine Research Journal2017. p. 1-.

56. Dodo M, Nagasaka K. Issues on Acceptance of Residents Rooting in Foreign Countries at Medical Institutions: Case of People from China Living in Yamanashi Prefecture (In Japanese). Bulletin of Faculty of Nursing, Yamanashi Prefectural University. 2013;15:1-9.

57. Umemura N, Martinez M, Hatashita H. Healthcare and daily needs of expectant Brazilian women residing in Japan (Analysis of fieldwork conducted during prenatal examinations and home visits) (In Japanese). Japanese Journal of Public Health. 2012;59(10):762-70.

58. Hashimoto H, Ito K, Yamaji Y, Sasaki Y, Murashima S, Yanagisawa S. Difficulties of pregnancy, delivery, and child raising for immigrant women in Japan and their strategies for overcoming them (In Japanese). Journal of International Health. 2011;26(4):281-93.

59. Nagata F, Hamai T, Sugata K. Issues regarding temporary interpreters when using medical services for Brazilians in Japan (In Japanese). Journal of International Health. 2010;25(3):161-9.

60. Sugiura K. Childrearing Brazilian women’s perceptions of perinatal care, the　healthcare system in Japan　and related sociocultural　　　　　　　　　　　　　　aspects: The third report (In Japanese). Japanese Journal of Maternal Health. 2009;50(2):267-74.

61. Itoi Y. Feeling with the Use of Medical Institution by Cambodian People Living in Japan and Their View on Health (In Japanese). Journal of Japan Society or Nursing Health Care. 2008;10(1):55-64.

62. Kubota K, Minamitani S, Tanaka Y. Current status and issues of perinatal care for Brazilians in Japan (Part 2) - Through the case of Brazilians in Japan (In Japanese). Japanese journal of maternal health. 2004;45(3):204.

63. Kitada A, Eto T, Nakaumura Y. Survey on childbirth and child-rearing among foreigners residing in Japan (In Japanese) The Journal of Child Health. 1993;52(2):201.

64. Lee S, Nguyen NHT, Takaoka S, Do AD, Shirayama Y, Nguyen QP, et al. A Study on the Health-Related Issues and Behavior of Vietnamese Migrants Living in Japan: Developing Risk Communication in the Tuberculosis Response. International Journal of Environmental Research and Public Health. 2023;20(12).

65. Paudel S, K C Bhandari A, Gilmour S, Lee HJ, Kanbara S. Barriers and facilitating factors to healthcare accessibility among Nepalese migrants during COVID-19 crisis in Japan: an exploratory sequential mixed methods study. BMC Public Health. 2023;23(1):1226-.

66. Morita N, Kanamori M, Nochi M, Kondo N. A mixed methods study on specifying the inhibitory factors to access medical services and effective support for foreign residents living in Japan (In Japanese). Journal of International Health. 2021;36(3):107-21.

67. Nagamatsu Y, Barroga E, Sakyo Y, Igarashi Y, Hirano O Y. Risks and perception of non-communicable diseases and health promotion behavior of middle-aged female immigrants in Japan: a qualitative exploratory study. BMC Women's Health. 2020;20:1-9.

68. Hong QN, Pluye P, Fàbregues S, Bartlett G, Boardman F, Cargo M, et al. Mixed methods appraisal tool (MMAT), version 2018. Registration of copyright. 2018;1148552(10).
