# Supplementary material for: Barriers and facilitators for healthcare access among immigrants in Japan: a mixed methods systematic review and meta-synthesis
Source: Lancet Reg Health West Pac. 2025 Jan 10;54:101276. doi: 10.1016/j.lanwpc.2024.101276 (PMC11774800; doi:10.1016/j.lanwpc.2024.101276)
Supplement: Suppl_protocol [file mmc2.docx]

1. **Review title.**

Barriers and facilitators for healthcare access among immigrants in Japan: A mixed methods systematic review and meta-synthesis

1. **Anticipated or actual start date.**

4/20/2023

1. **Anticipated completion date.**

9/1/2024

1. **Stage of review at the time of this submission.**


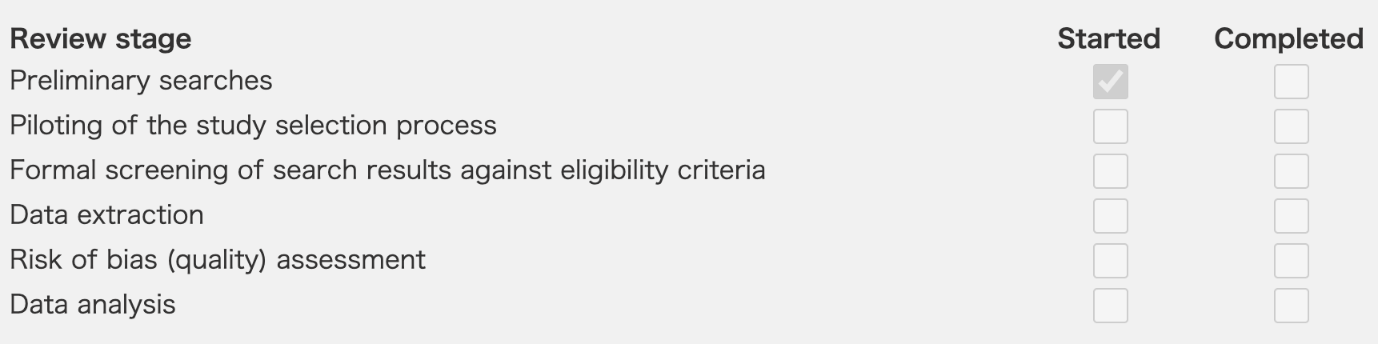


1. **Named contact:** Nobutoshi Nawa (Dr. Nawa)
2. **Named contact email:** [nawa@ioe.tmd.ac.jp](mailto:nawa@ioe.tmd.ac.jp)
3. **Named contact address:**
4. **Named contact phone number.**

Department of Global Health Promotion, Tokyo Medical and Dental University (TMDU), 1-5-45, Yushima, Bunkyo-ku, Tokyo 113-8519, Japan

1. **Organisational affiliation of the review.**

None

1. **Review team members and their organisational affiliations.**

**Mandatory**

1. Dr. Yu Par Khin, Tokyo Medical and Dental University (TMDU)
2. Dr. Floret Maame Owusu, Tokyo Medical and Dental University (TMDU)
3. Dr. Nobutoshi Nawa, Tokyo Medical and Dental University (TMDU)
4. Dr. Takeo Fujiwara, Tokyo Medical and Dental University (TMDU)
5. **Funding sources/sponsors.**

None

1. **Conflicts of interest.**

None

1. **Collaborators.**

None

1. **Review question.**

What are the barriers and facilitators to healthcare access among migrants in Japan?

Objective 1: What are the barriers and facilitators for healthcare access of immigrants in Japan?

1. **Searches**

We will conduct a literature search using the following databases: PubMed, Cumulative Index to Nursing and Allied Health Literature (CINAHL), and Web of Science Core Collection. We will also search the Japanese database, Ichushi. The search will be conducted in April 2023. We will also perform an updated search on PubMed, CINAHL, Web of Science Core Collection, and Ichushi on January 2024, using the same search terms. The publication year will not be limited. No search filters, such as article type, or language will be applied. We will also perform a manual search of the reference list of relevant articles and consult with experts to ensure that all relevant articles are identified. Additionally, a Google search was performed for grey literature using both English and Japanese search terms.

1. **URL to search strategy.**

All search dates and search terms used will be detailed in the Supplementary file. Thus, we will upload the Supplementary file.

Give a link to the search strategy or an example of a search strategy for a specific database if available (including the keywords that will be used in the search strategies).

Alternatively, an electronic file could be supplied which will be linked to the Register record. This will be made publicly available from the published record immediately, or it can be held in confidence until the review has been completed, at which time it will be made publicly available.

1. **Condition or domain being studied.**

Barriers and facilitators for healthcare access

1. **Participants/population.**

Immigrants currently residing in Japan with a residential (visa) status of more than three months (eligible for the National Healthcare scheme).

1. **Intervention(s), exposure(s).**

Migration status

1. **Comparator(s)/control.**

None

1. **Types of study to be included.**

Studies will be included if they reported the healthcare access barriers and facilitators of migrants in Japan with or without comparison with the Japanese. Both qualitative and quantitative studies will be included. Studies will be excluded if they reported the health status of the migrants but not included healthcare access.

1. **Context**

None

1. **Main outcome(s).**

Barriers and facilitators to healthcare access among Migrants in Japan

1. **Measures of effect**

Descriptive narration

1. **Additional outcome(s).**

Overall health status and consequences of barriers to healthcare access among migrants in Japan

1. **Measures of effect**

Descriptive narration

1. **Data extraction (selection and coding).**

Study selection

Two independent researchers (YPK, FMO) will review the study titles and abstracts. If a study meets the selection criteria, the full text will be retrieved for further evaluation. Disagreement between the two researchers will be resolved by discussion and a third author (NN or TF) will weigh in to reach a consensus if needed. Mendeley and Rayyan will be used for reference management.

Data extraction

Data extraction will include information on sample size, study authors, the region where the study was conducted, year of publication, study design, type of healthcare access, presence of comparison as well as participant characteristics (age, sex, nationality, socio-demographic factors (e.g. education level, income), residential (visa) status, length of stay in Japan, Japanese language proficiency, presence or absence of family in Japan, presence of chronic diseases, key conclusions and recommendations of the study, and potential conflicts of interest of the authors.

1. **Risk of bias (quality) assessment.**

The quality of each study will be assessed using mixed method appraisal tools (MMAT). One researcher (YPK) will assess the risk of bias (quality) and the second author (FMO) will check the assessment. Disagreement between the two researchers will be resolved by discussion and a third author (NN or TF) will weigh in to reach a consensus if needed.

1. **Strategy for data synthesis.**

PRISMA flowchart will be applied to synthesize the data frame. As our extracted data will be diverse in the methodology as well as the type of healthcare, we will use the descriptive-analytic method (Arksey and O'Malley, 2005) in narration. We will construct a flowchart to involve all the conclusions from the studies.

1. **Analysis of subgroups or subsets.**

Subgroup analyses for the barriers to healthcare access will be done for the following if extractable.

1. With the type of healthcare access
2. **Type and method of review**

Epidemiologic, Health inequalities/health equity, Narrative synthesis, Service delivery, Systematic Review.

1. **Language.**

English.

1. **Country.**

Japan

1. **Other registration details.**

Blank

1. **Reference and/or URL for published protocol.**

Blank

1. **Dissemination plans.**

A paper will be submitted to a leading journal in this field.

1. **Keywords.**

foreign nationalities; healthcare; healthcare access; Japan; migrants; systematic review; Japan

1. **Details of any existing review of the same topic by the same authors.**

Not applicable

1. **Current review status.**

Review Ongoing

1. **Any additional information.**

None
